# Spotting the enemy within: Targeted silencing of foreign DNA in mammalian genomes by the Krüppel-associated box zinc finger protein family

**DOI:** 10.1186/s13100-015-0050-8

**Published:** 2015-10-02

**Authors:** Gernot Wolf, David Greenberg, Todd S. Macfarlan

**Affiliations:** The Eunice Kennedy Shriver National Institute of Child Health and Human Development, The National Institutes of Health, Bethesda, MD 20892 USA; The Gladstone Institute of Virology and Immunology, University of California, San Francisco, CA 94158 USA; Present address: Pacific Biosciences, 1380 Willow Road, Menlo Park, CA 94025 USA

**Keywords:** KRAB zinc finger proteins, Transcription factors, Endogenous retroviruses, Transposable elements, Epigenetic silencing, Adaptive evolution

## Abstract

Tandem C2H2-type zinc finger proteins (ZFPs) constitute the largest transcription factor family in animals. Tandem-ZFPs bind DNA in a sequence-specific manner through arrays of multiple zinc finger domains that allow high flexibility and specificity in target recognition. In tetrapods, a large proportion of tandem-ZFPs contain Krüppel-associated-box (KRAB) repression domains, which are able to induce epigenetic silencing through the KAP1 corepressor. The KRAB-ZFP family continuously amplified in tetrapods through segmental gene duplications, often accompanied by deletions, duplications, and mutations of the zinc finger domains. As a result, tetrapod genomes contain unique sets of KRAB-ZFP genes, consisting of ancient and recently evolved family members. Although several hundred human and mouse KRAB-ZFPs have been identified or predicted, the biological functions of most KRAB-ZFP family members have gone unexplored. Furthermore, the evolutionary forces driving the extraordinary KRAB-ZFP expansion and diversification have remained mysterious for decades. In this review, we highlight recent studies that associate KRAB-ZFPs with the repression of parasitic DNA elements in the mammalian germ line and discuss the hypothesis that the KRAB-ZFP family primarily evolved as an adaptive genomic surveillance system against foreign DNA. Finally, we comment on the computational, genetic, and biochemical challenges of studying KRAB-ZFPs and attempt to predict how these challenges may be soon overcome.

**Electronic supplementary material:**

The online version of this article (doi:10.1186/s13100-015-0050-8) contains supplementary material, which is available to authorized users.

## Introduction

Tandem C2H2-type zinc finger proteins (ZFPs) make up the single largest transcription factor family in mice and humans with approximately 600 and 700 genes, respectively [1]. The largest of several ZFP subtypes are the Krüppel-associated box (KRAB) domain–containing ZFPs, called KRAB-ZFPs in mice and KZNFs in humans (hereafter all referred to as KRAB-ZFPs), with estimates of approximately 200 and 300 genes in mice and humans, respectively [1, 2].

KRAB-ZFPs contain a potent KRAB repression domain and tandem arrays of zinc fingers (ZNFs) that mediate DNA binding. What makes KRAB-ZFPs exceptional among other DNA binding transcription factors is their ability to bind to long stretches of DNA by combinatorial use of up to several dozen ZNFs that serve as modular DNA binding units. These exceptional modular DNA binding properties were co-opted for use in gene-editing applications, forming the basis of the first generation of engineered sequence-specific DNA modifying enzymes called zinc finger nucleases [3, 4]. However, the natural target sites of mammalian KRAB-ZFPs are largely unknown. Importantly, some of the characterized KRAB-ZFPs are associated with metabolism, differentiation, apoptosis, and other cellular functions [2, 5], but overall very few KRAB-ZFPs have been functionally investigated.

KRAB-ZFPs are also unique among transcription factor families in that a large fraction of their members have DNA binding domains that are rapidly evolving. This rapid evolution may contribute to morphological and behavioral evolution by controlling expression of developmental genes [2, 6]. However, recent experimental and computational discoveries have provided compelling evidence that perhaps a large proportion of evolutionarily young KRAB-ZFPs function as part of a surveillance system that protects mammalian genomes from infectious retroviruses, their endogenous counterparts, and nonretroviral retrotransposons [7–11]. In this hypothesis, the species-specific amplification and diversification of mammalian KRAB-ZFPs are consequences of ancient and ongoing germ-line colonization events by mobile DNA elements. Here we will summarize recent progress that implicated KRAB-ZFPs as molecular guardians of genomic integrity and discuss the possible connections between anti-mobile DNA KRAB-ZFPs and those that have evolved to fulfill functions beyond genomic defense.

## Review

### Evolution and function of the KRAB-ZFP family

#### Origins of the KRAB-ZFP family

KRAB-ZFPs are believed to have evolved from the Meisetz (PRDM9) gene [12], which has KRAB and SET domains and a tandem array of C2H2 ZNFs. Meisetz homologues have been identified in sea urchins and tunicates, indicating that the ancestral KRAB domain arose before the common deuterostome ancestor of vertebrates and echinoderms at least 520 million years ago [12]. However, unlike most mammalian KRAB-ZFPs, which interact with KAP1 (also known as TRIM28 or TIF1β) and are therefore potential transcriptional repressors, Meisetz acts as an H3K4 methyltransferase through its SET domain [13, 14]. Thus the ancestral KRAB domain might have acted as a transcriptional activator instead of a repressor [12, 15] and changes in the KRAB domain or the evolution of new co-repressors may have facilitated KRAB-ZFPs to function as repression factors. Indeed, KRAB domains evolved rapidly [16], and the TRIM family, to which KAP1 belongs, is highly diversified in vertebrates [17]. Although it is not known when KRAB-ZFPs began recruiting KAP1 or possibly other KAP1-like co-repressors, it seems that a Meisetz-derived KRAB-ZFP lost its SET domain at some time during evolution. Functional changes in the KRAB domains and/or KAP1 may then have resulted in a novel, highly specific transcriptional repression factor that rapidly amplified and diversified throughout tetrapod evolution.

KRAB-ZFPs with a SCAN domain have been found in mammals and lizards but are absent in frog and chicken, indicating that this domain was acquired in KRAB-ZFPs around the root of the amniote branch but subsequently got lost in some species [18]. Interestingly, the SCAN domain shows striking homology to the C-terminal portion of the gag capsid protein from the Gmr1-like family of Gypsy/Ty3-like LTR retrotransposons. It was therefore hypothesized that a retrotransposon insertion into a KRAB-ZFP gene resulted in the exaptation of this domain [18]. However, the biological function of SCAN domains in mammalian KRAB-ZFPs is entirely unknown.

#### DNA binding and initiation of epigenetic silencing

**Fig. 1:**
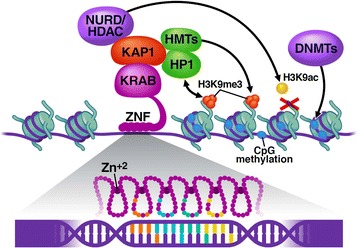
Model of KRAB-ZFP binding to DNA and induction of heterochromatin formation. Protein–DNA interaction between ZNFs and DNA are mainly mediated by four amino acids at positions −1, 2, 3, and 6 of the α-helix (colored circles). KAP1 is recruited through the KRAB domain and interacts with the NURD/HDAC repressor complex and histone methyltransferases (HMTs) (e.g. SETDB1), which catalyze the removal of H3K9ac and the addition of H3K9me3, respectively. HP1γ interacts with both KAP1 and H3K9me3. DNA methyltransferases (DNMTs) methylate genomic CpG sites, leading to inheritable silencing

To date, the vast majority of KRAB-ZFP research has focused on human and mouse KRAB-ZFPs. Therefore, it is important to point out that the findings from these studies may not always be applicable to tetrapod KRAB-ZFPs in general. Nevertheless, all KRAB-ZFPs contain tandem arrays of up to 36 C2H2-type ZNFs, usually encoded by a single exon at the 3’ end of the gene [2, 6, 8]. Each ZNF directly interacts with three consecutive nucleotides and one nucleotide of the reverse-complement strand within the adjacent trinucleotide (Fig. 1). The amino acids mainly responsible for the DNA interaction, and therefore the binding specificity of ZNFs, are located at positions −1, 2, 3, and 6 of the DNA-contacting alpha helix. The looped structure of the ZNF is stabilized by a zinc ion that is characteristically contacted by two cysteine and histidine residues [19] (Fig. 1). Unlike most transcription factors that bind rather short DNA sequences, KRAB-ZFPs can use their tandem ZNF array structure to specifically target large stretches of DNA that are unlikely to be found in significant numbers in the genome by chance. On average, mouse and human KRAB-ZFPs have about eight ZNFs, thus the average KRAB-ZFP target motif is expected to have about 24 nucleotides [1]. However, it has been argued that not all ZNFs of a single KRAB-ZFP are necessarily involved in DNA binding [20–22]. For example, Gli, a non-KRAB containing ZNF protein, uses only four of its five ZNFs to interact with DNA [23], and ZNF91, one of the largest known KRAB-ZFP with 36 ZNFs, mainly uses the 11 most N-terminal ZNFs to bind efficiently to its genomic targets, with some 12 C-terminal ZNFs being dispensable for DNA-recognition [8]. Furthermore, CTCF, a KRAB-less tandem-ZFP with 11 ZNFs was shown to bind to various motifs via clustering its ZNFs in several combinations [24].

Several DNA binding prediction models for tandem-ZFPs have been developed by using bacterial one-hybrid systems, empirical calculations of pairwise amino acid–nucleotide interaction energies, and knowledge from the X-ray crystal structure of a three-fingered, C2H2-type ZFP (Zlf268 or Egr1) [4, 9, 25–27]. However, even empirically based tandem-ZFP binding predictions generally rely on data gained by testing the DNA binding preferences of individual ZNFs in heterologous hybrid proteins and in a nonchromosomal context, which may not always reflect their true DNA binding specificity. Furthermore, amino acids of ZNFs other than the four “specificity residues” can influence binding specificity [9] and ZNF “context” may contribute to a given finger’s preferred binding site. In addition, some ZNFs within an array may not interact with DNA and the DNA sequence flanking the binding motif can interfere with ZNF binding [24]. Moreover, certain ZNFs bind specifically to methylated DNA [28, 29], indicating that epigenetic modifications can also influence DNA binding of ZFPs. Therefore, it will be very difficult if not impossible to ever reliably predict genomic tandem-ZFP binding sites without experimental testing. Nevertheless, solving the crystal structure of several large KRAB-ZFPs bound to DNA would be a technical milestone that may be necessary to help improve existing models.

About 30–40 % of mammalian tandem-ZFPs have a KRAB domain [1] that, in many but not all cases, recruits the corepressor KAP1 [15, 30–32]. KAP1 compacts chromatin through recruiting histone-modifying factors, such as the NuRD histone deacetylase (HDAC) complex and the histone methyltransferase (HMT) SETDB1, which remove transcription-promoting histone acetylation and add the repressive histone 3 lysine 9 trimethylation (H3K9me3) mark, respectively [33, 34] (Fig. 1). During early embryogenesis, reporter genes that have been silenced by artificially tethered KAP1 or endogenous KRAB-ZFPs remain transcriptionally repressed through DNA methylation, even after the reporter gene has been released from KAP1 [35, 36]. This indicates that KRAB/KAP1-induced silencing is epigenetically heritable when initiated in early embryos. KAP1-induced heritable silencing is partially facilitated by heterochromatin protein 1 (HP1), which is recruited by KAP1 through its PxVxL motif [37] and interacts with DNA methyltransferases [38] (Fig. 1). Moreover, KRAB/KAP1-induced heterochromatin can spread over large distances through self-promoting mechanisms, which allows epigenetic silencing beyond the initiation site [39]. Additional corepressors implicated in KAP1/SETDB1-dependent silencing include hnRNP K [40], CAF-1 [41], ATRX/DAXX [42] and the human silencing hub (HUSH) complex [43].

#### Expansion and diversification of KRAB-ZFP genes

Tandem-ZFP genes are predominantly organized in genomic clusters [2, 6]. For instance, about one-third of all human tandem-ZFP genes are located within six clusters on chromosome 19, the largest one containing 72 tandem-ZFP genes within a 3.5-Mb region [1]. Generally, human tandem-ZFPs that are located in the same cluster also group together phylogenetically, indicating that they result from local gene duplication events [6]. Through chromosomal translocations and other genomic rearrangements new tandem-ZFP genes can gradually disperse and act as seeds for new clusters [1, 6]. Interestingly, the chromatin landscape of KRAB-ZFP clusters is distinct from the rest of the genome. A recent method (in situ Hi-C) generated a 3D map of the human genome and correlated this information with epigenetic marks. This analysis revealed that KRAB-ZFP clusters possess a unique chromatin organization, consisting of both active (e.g. H3K36me3) and repressive H3K9me3 histone modfications [44]. These findings were consistent with two previous studies: the first finding enriched levels of HP1 at the 3’ ends of KRAB-ZFP genes [45], and the second demonstrating a combination of low CpG density in gene bodies together with H3K9me3 and H3K36me3 at KRAB-ZFP loci [46]. Interestingly, the human KRAB-ZFP ZNF274 is itself responsible for H3K9me3 enrichment at the ZNF regions of KRAB-ZFP genes [47]. It has been speculated that the recruitment of KAP1 and H3K9me3 to the 3’ end of KRAB-ZFP genes (and perhaps more broadly the unique combination of histone marks over KRAB-ZFP clusters) protects against ectopic, non-allelic homologous recombination to some degree [47, 48]. Nevertheless, tandem-ZFP gene duplications occurred frequently over evolutionary time scales and resulted in several hundreds of KRAB-ZFP genes in mammals. This rapid amplification is likely catalyzed by the repetitive ZNFs of these genes, which are prone to illegitimate recombination and replication slippage [1, 49]. Thus, a fine evolutionary balance has been reached between the need for evolvability of KRAB-ZFPs, and the need to prevent loss of important KRAB-ZFPs by recombination events.

Although the KRAB domains and amino acids required for ZNF structure are generally well conserved amongst mammalian KRAB-ZFPs, positive selection at the residues that confer DNA binding specificity is common, especially between recently duplicated gene pairs [1, 49–51]. Thus, once a KRAB-ZFP gene duplicates, one of the pair may keep its ZNF structure to fulfill its original function, whereas the daughter gene becomes available to alter its DNA binding specificity and potentially gain new functions.

#### Why are there so many KRAB-ZFP genes in mammals?

Although the progenitor of the KRAB domain apparently dates to the last common deuterostome ancestor of chordates and echinoderms [12], KRAB-ZFPs are with few exceptions restricted to tetrapod vertebrates and are most abundant in mammals [1, 52]. Interestingly, the KRAB domain of KRAB-ZFPs has changed significantly during tetrapod evolution. A computational analysis of vertebrate KRAB domains revealed that in chicken, lizard and frog KRAB-ZFPs, some of the amino acids that are essential for KRAB-KAP1 interaction in mammals are not conserved [15, 31]. It is therefore unclear whether KRAB-ZFPs in these species can recruit KAP1. Furthermore, some of the oldest mammalian KRAB-ZFPs do not interact with KAP1 [15, 31], and some function as transcriptional activators instead [53, 54]. Thus, structural changes to the KRAB domain at some point during tetrapod evolution may have caused KRAB-ZFPs to recruit KAP1, establishing a new class of epigenetic repressors that subsequently rapidly amplified. However, experimental testing of interactions between nonmammalian KRAB domains and KAP1 and possibly other factors will be required before such a conclusion can be drawn with certainty. Interestingly, tandem-ZFPs with other domains than KRAB have expanded via gene duplications in insects and amphibians [55, 56]. This indicates that lineage-specific tandem-ZFP amplification and diversification is not restricted to KRAB-ZFPs.

Some KRAB-ZFPs have been associated with metabolism, differentiation, apoptosis, and human diseases [2, 5, 57, 58], but in most cases their genomic binding sites are unknown. Since the majority of KRAB-ZFPs are predicted to interact with KAP1, most KRAB-ZFPs are believed to repress transcription. Indeed, one of the first genome-wide studies of KRAB-ZFP DNA binding identified binding sites for ZNF263, a human KRAB-ZFP with a SCAN domain, near gene promoters. Importantly, ZNF263 knockdown derepressed a subset of ZNF263-targeted genes [59].

The rapid amplification and diversification of KRAB-ZFPs in tetrapods and especially mammals suggest that the bulk of recently emerged KRAB-ZFPs are involved in functions specific to these animals. Although a recent analysis of transcription factor expression during human fetal development demonstrated that the KRAB-ZFP family generally displays less tissue-specific expression levels than other transcription factor families (Siebenthall, K.T., *personal communication*), a fraction of KRAB-ZFPs are differentially expressed in adult tissues [60]. Notably, many KRAB-ZFPs are highly expressed in evolutionarily recent tissues, such as the mammalian-specific placenta [15]. Mammals might also require a large number of KRAB-ZFPs to control mammal-specific innovations in processes such as erythropoiesis [61] and development of the adaptive immune system [62]. Since even closely related species such as higher primates differ in their KRAB-ZFP arsenal, it was suggested that KRAB-ZFPs may also contribute to human brain development [63]. Interestingly, the transcriptional activity of certain KRAB-ZFP orthologues greatly varies between human and chimpanzee brain, suggesting that KRAB-ZFPs may change their expression levels after speciation to adapt to new functions [51, 63]. While the increase in the complexity of mammalian development might explain some of the KRAB-ZFP diversification, the number of KRAB-ZFPs does not correlate with brain size or the duration of embryonic development [64]. According to a recent study, opossums have nearly twice as many KRAB-ZFP genes as humans [15]. Indeed, recent findings support the hypothesis that the majority of KRAB-ZFPs function as repressors of parasitic DNA rather than as conventional gene-regulating transcription factors. Moreover, many KRAB-ZFPs might regulate genes through targeting nearby remnants of parasitic DNA that has been co-opted as novel regulatory sequences. In the following section, we will briefly discuss the impact of parasitic DNA elements on mammalian evolution and review recent findings suggesting that those elements triggered KRAB-ZFP expansion and diversification.

### KRAB-ZFPs are adaptive repressors of foreign DNA

#### Retrotransposons and the need for an adaptive repression system

Retroviruses have been invading mammalian germ lines for millions of years, accumulating in the form of endogenous retroviruses (ERVs) that account for approximately 8 % of the human genome [65]. Mammalian genomes also contain many nonretroviral retrotransposons—long interspersed nuclear elements (LINE) and short interspersed nuclear elements (SINEs)—that cannot form infectious particles but amplify through retrotransposition in host cells.

Growing evidence supports an important role of ERVs and nonretroviral retrotransposons, both also referred to as endogenous retroelements (EREs), in certain developmental processes through host co-option of viral proteins and regulatory sequences [66–74]. However, uncontrolled EREs are a threat to the genomic integrity of the host organism. In mice, several active ERV groups contribute to an estimated 10 % of all *de novo* mutations [75, 76]. Although no replication-competent human ERVs (HERVs) have been described yet, HERV fragments are associated with human lymphomas and other cancer types, and HERV-K particles were detected in human pre-implantation embryos [70, 77–79]. The HERV-K subgroup HML2 is responsive to the HIV-1 transactivator protein (Tat) [80], and some of these elements encode functional envelope and integrase proteins. HERV-K envelope proteins can be incorporated into HIV particles [81] and may be a biomarker for HIV latency [82]. Furthermore, the long terminal repeats (LTRs) of HERV-K contain many binding sites for inflammatory transcription factors, suggesting that these ERVs contribute to the pathology of inflammatory disease [83]. Finally, several non-retroviral retrotransposons have been linked to many human diseases [84–87], and it has been speculated that LINEs decrease longevity by eroding genomic integrity [88]. These studies highlight the potentially damaging effects of uncontrolled activation of retrotransposons.

To defend their genomes against exogenous retroviruses and EREs, mammals rely on a wide range of defense mechanisms, including APOBEC proteins [89], PIWI-interacting RNAs (piRNAs) [90], nucleic acid sensors [91], and transcriptional repression [92–94]. EREs are transcriptionally repressed by stable epigenetic silencing that can be maintained through cell division. This silencing mechanism requires distinct and partially overlapping machinery in pluripotent and somatic tissues. In somatic tissues, EREs are repressed by DNA methylation, as revealed by mutations in DNA methyltransferases [95–97]. In pluripotent embryonic stem cells (ESCs), ERVs and LINEs are repressed primarily by machinery that creates repressive histone modifications, most notably H3K9me3 [98], whereas DNA methylation is largely dispensable [99, 100]. However, histone modifications established in early development seem to be required to initiate or stabilize heritable DNA methylation at EREs during differentiation and development [36, 101].

Although epigenetic repression of retroviral DNA in ESCs has been the subject of numerous studies, the factors that target epigenetic silencing machinery to EREs in mammals have remained elusive. One strategy developed by eukaryotes to cope with mobile DNA diversity relies on short RNAs that are expressed by the transposons themselves and allow guiding of the silencing machinery to the expressed element through base pairing [102]. These short RNAs include small interfering RNAs (siRNAs) that regulate LTR transposons in yeast [103], siRNAs that target DNA methylation at heterochromatin in plants [104], and piRNAs that guide silencing in animal germ cells [90, 105–108]. Tetrapods likely employ an additional and equally important strategy to recognize and silence EREs: genetic encoding of an army of evolutionarily selected DNA-binding transcription factors. To silence EREs with minimal off-target effects, such transcription factors need to be able to bind large DNA motifs that are unlikely to appear in the genome by chance. Furthermore, these factors need to be evolutionarily adaptable to recognize newly emerging EREs and possess a potent repression domain to stably silence these elements. The KRAB-ZFP family alone fulfills all these criteria. Indeed, while small RNAs may be the predominant way to target EREs in plants and mammalian germ cells, mammals seem to rely on KRAB-ZFPs to recognize and silence retroviruses and EREs during early embryonic development.

#### KRAB-ZFPs repress exogenous and endogenous retroviruses

The most compelling direct evidence that KRAB-ZFPs repress retroviruses and EREs comes from the identification of the ZFP809/KAP1 murine leukemia virus (MuLV) repression complex and from two loss-of-function studies of the KRAB-ZFP corepressors KAP1 and SETDB1 in ESCs that revealed ERV activation phenotypes.

It has been long known that a multi-component repressor complex binds to a 17-bp sequence within the proline tRNA primer binding site (PBS^pro^) of integrated MuLV in murine pluripotent stem cells [109, 110]. The identification of KAP1 as an integral component of the PBS^pro^ targeting repressor complex [111] strongly implied that a KRAB-ZFP is the DNA binding factor that tethers the complex to MuLV. Indeed, ZFP809, a mouse KRAB-ZFP with no human orthologue, was subsequently identified as the recognition module that targets the PBS^pro^ and recruits KAP1 [112]. Shortly thereafter, genetic removal of KAP1 or its interacting protein SETDB1 revealed a broad requirement for these proteins in heterochromatin formation and ERV silencing in ESCs and in viability [36, 100, 113]. However, knockout of KAP1 in murine embryonic fibroblasts (MEFs) does not affect ERV expression [113], consistent with the observation that KAP1 repression during early embryogenesis leads to irreversible silencing that is maintained by DNA methylation and does not persistently require KAP1 [35]. These studies thus laid the foundation supporting the KRAB-ZFP family as the likely candidate for ERV recognition and transcriptional silencing.

Direct evidence linking an individual KRAB-ZFP to ERE silencing came from genome-wide binding and genetic knockout studies of ZFP809. In ESCs, ZFP809 binds to several PBS^pro^ containing ERVs and recruits the KAP1/SETDB1 repressor complex to these elements. Moreover, ZFP809 knockout leads to a strong reactivation of VL30 elements with a PBS^pro^ (VL30^Pro^) in postimplantation embryos and in most organs and tissues of adult animals [7]. Interestingly, VL30^Pro^ elements are inactive in pre-implantation embryos and ESCs even in the absence of ZFP809, most likely because certain transcription factors are missing in these embryos/cells. Nevertheless, ZFP809 is required to initiate epigenetic silencing of these elements in ESCs to prevent ERV reactivation during differentiation. Once silenced by ZFP809 in ESCs, VL30^Pro^ remain transcriptionally silent in differentiated cells, even when ZFP809 is no longer present [7]. These findings support the model in which KRAB/KAP1 silencing is initiated in early embryos and heritably maintained in somatic tissues without a continuous requirement for KRAB-ZFPs or KAP1 [35].

However, KAP1 is also required for ERV silencing in neural progenitor cells, indicating that ERV repression by KRAB/KAP1 is not strictly restricted to ESCs [114]. Moreover, SETDB1 appears to be continuously required to maintain ERV silencing in some differentiated cell types, as conditional SETDB1 deletion in MEFs and B lymphocytes leads to massive reactivation of several ERV groups [7, 115]. Importantly, different groups of ERVs become reactivated in SETDB1 knockout ESCs and B lymphocytes, indicating that not only the loss of repressive chromatin marks but also the presence of possibly tissue-specific transcription factors determines which ERVs become de-repressed [115].

Although ZFP809 is so far the only KRAB-ZFP whose role in ERV silencing is supported by convincing biochemical and genetic evidence, several other KRAB-ZFPs have been implicated (Table 1). For example, ZFP819 knockdown led to a significant upregulation of IAP ERVs and other EREs in murine ESCs. Although a defined target motif for ZFP819 has not been identified in these elements, overexpression of ZFP819 also inhibits expression of a luciferase reporter containing an IAP LTR fragment [116].

**Table 1:** KRAB-ZFPs reported to bind to exogenous/endogenous retroviruses and other EREs

KRAB-ZFP	Organism	Target	Motif	Supporting evidence	Reference
ZFP809	Mouse	MuLV, VL30	18 bp	ChIP-seq, KO, Rep, EMSA	[7], [112]
ZFP819	Mouse	IAP, LINE	n.d.	ChIP, KD, Rep	[116]
Gm6871	Mouse	LINE	18 bp	ChIP-seq, KD, Rep	[10]
ZNF350	Human	HIV	21 bp	ChIP, Rep	[122]
ZNF175	Human	HIV	11/17 bp	Rep	[121]
ZNF10	Human	HIV	n.d.	Rep, KD, EMSA	[123]
ZNF282	Human	HTLV	8 bp	Rep, EMSA	[53]
ZNF91	Human	SVA	>60 bp	Rep	[8]
ZNF93	Human	L1	51 bp	ChIP-seq, Rep	[8]
Various ZNFs	Human	Various EREs	9-18 bp	ChIP-seq	[9]

*KO* genetic knockout studies, *Rep* reporter assays, *KD* knockdown by siRNA, *EMSA* electrophoretic mobility shift assays, *n.d.* not determined

Several lines of evidence suggest that human ERVs (HERVs) are repressed by KRAB-ZFPs. KAP1 is enriched at Class I and II HERVs in human ESCs and although the KRAB-ZFPs that recruit KAP1 to these elements have not been identified, a 39-bp sequence was demonstrated to be critical for KAP1-dependent silencing of HERV-K elements in reporter assays [11]. Intriguingly, this sequence overlapped with the PBS of these ERVs, which is complementary to a human lysine tRNA. Moreover, many human KRAB-ZFPs interact with specific ERV classes when overexpressed as GFP-fusion proteins in 293 T cells [9]. However, none of these interactions were validated by other types of binding or functional assays. Thus it is premature to conclude that all these proteins are indeed ERV silencers.

#### KAP1/KRAB-ZFP repression of nonretroviral retrotransposons

The first indirect evidence that KRAB-ZFPs also repress nonretroviral EREs came from two studies that investigated the genome-wide binding patterns of KAP1 in human ESCs and primary human T lymphocytes [10, 11]. These studies showed that KAP1 is bound to a defined subset of LINE-1 (L1) transposons and several groups of SINE-VNTR-Alu (SVA) elements. The identified target sequences in these elements induced epigenetic silencing of reporter genes in human ESCs. Furthermore, the murine KRAB-ZFP Gm6871 was identified as a L1 binding protein by ChIP-seq [10].

Shortly thereafter, it was shown that several human- and primate-specific SVA and L1 elements are de-repressed in trans-chromosomic murine ESCs that contain a copy of human chromosome 11 [8]. This indicated that the repression factors controlling these elements in humans are absent in mice. In a subsequent screen of a selection of 14 highly expressed primate-specific KRAB-ZFPs that appeared in the catarrhine lineage 25–35 million years ago, ZNF91 and ZNF93 repressed reporter constructs containing SVA and L1PA-type retrotransposons, respectively. Overexpression of these KRAB-ZFPs in trans-chromosomic murine ESCs resulted in re-silencing of their target elements. Furthermore, ChIP-seq confirmed ZNF93 binding to endogenous L1PA elements, providing conclusive evidence that human KRAB-ZFPs bind and repress retrotransposons [8].

Notably, no KRAB-ZFPs that target DNA transposons have been identified to date. This is not surprising since DNA transposons only constitute a small fraction of mammalian genomes. Furthermore, DNA transposons are generally not active in mammals and repression of these elements by KRAB-ZFPs might therefore be unnecessary.

#### A potential role of KRAB-ZFPs in repressing HIV, HTLV-1, and nonviral transgenes

Several years before KAP1 and KRAB-ZFPs were identified as ERE repressors, a handful of studies explored the use of artificially designed KRAB-ZFPs to restrict HIV infection. These artificial KRAB-ZFPs successfully repressed HIV transcription by binding to the proviral LTR or PBS [117–119], showing that KRAB-ZFPs can be “designed” to repress a virus of interest. However, this strategy has not been adapted for clinical applications.

Intriguingly, at least three human KRAB-ZFPs, ZNF175 (OTK18) [120, 121], ZNF350 (ZBRK1) [122] and ZNF10 [123], were associated with transcriptional repression of the HIV LTR. Furthermore, ZNF282 (HUB1) reportedly binds an 8-bp sequence in the human T-cell leukemia virus (HTLV) LTR and represses transcription from the viral promoter [53]. Interestingly, HTLV repression was not dependent on the ZNF282 KRAB domain, which surprisingly activated transcription. An unidentified domain of ZNF282 may therefore be responsible for HTLV repression [53].

Retrovirus and ERE repressing KRAB-ZFPs are generally believed to evolve when a species is continuously exposed to these elements [8–10, 64]. The identification of these four human KRAB-ZFPs as repressors of lentiviruses is therefore somewhat surprising because ZNF10, ZNF175, ZNF350 and ZNF282 (unlike ZFP809, ZNF91, and ZNF93) are well conserved in mammals. In fact, ZNF282 is one of only three human KRAB-ZFPs that have orthologues in nonmammalian amniotes [15]. Although lentiviruses have been infecting primates for millions of years [124], humans have not been exposed to HIV for more than a few decades and the HIV-related simian immunodeficiency virus was estimated to be only 32,000 years old [125]. Similarly, although HTLV is believed to have infected humans for tens of thousands of years [126] and HTLV-related simian T-cell leukemia virus (STLV) strains have been found in nonhuman primates [127], HTLV clearly emerged after ZNF282. However, it cannot be excluded that these conserved KRAB-ZFPs originally evolved to repress ancient lentiviruses or lentivirus-like elements and therefore still recognize current HIV and HTLV strains.

Although there is some evidence that ZNF175 expression is correlated with HIV infection [128, 129], it is unclear whether any of the KRAB-ZFPs mentioned above inhibit HIV or HTLV *in vivo*. The HIV and HTLV LTRs may simply contain sequences that resemble endogenous targets of these KRAB-ZFPs and binding to these viral sequences is not evolutionarily intended or of any biological consequence. Indeed, the HIV-1 LTR sequence that is necessary for ZNF10-mediated repression overlaps with NF-κB and Sp1 binding sites [123] which are commonly found in gene promoters. Furthermore, ZNF350 was identified as a tumor-suppressor gene [130–133], ZNF282 was associated with cancer progression [134, 135], and ZNF175 might play a role in neuronal survival [136].

Thus, these ancient KRAB-ZFPs may have functions other than retroviral restriction. The question remains why HIV and HTLV did not mutate to evade these potential repressor proteins. Possibly, transcriptional repression by KRAB-ZFPs is advantageous for these viruses under some circumstances (e.g., establishment of latency). Therefore, HIV and other viruses might have actually evolved to be bound by certain KRAB-ZFPs.

Surprisingly, a murine KRAB-ZFP has been associated with silencing of a bacterial transgene in mice. A 0.9-kb fragment of the bacterial xanthine–guanine phosphoribosyltransferase (gpt) gene is rapidly silenced by DNA methylation and histone modifications in mice of some strains (e.g., C57BL/6) but not others (e.g., DBA/2) [137, 138]. Breeding mice carrying the unmethylated transgene to mice that methylate it leads to transgene methylation, indicating that a dominant factor, present only in some mouse strains, is required for silencing. This factor, initially named strain-specific modifier 1, was later identified as the KRAB-ZFP gene 2610305D13Rik [139]. Indeed, the observation that silencing commences during implantation and that DNA methylation spreads into neighboring regions is consistent with KRAB-ZFP-mediated silencing [137, 140]. However, direct binding of this KRAB-ZFP to the transgene has not been shown. Furthermore, It is unclear what the genomic targets of 2610305D13Rik are or whether the gpt-containing transgenic sequence resembles an ERE or another genomic target [139].

#### An evolutionary arms race between KRAB-ZFPs and EREs

Nonretroviral transposable elements such as SINEs and LINEs are vertically transmitted from generation to generation, allowing the host to evolve repressive mechanisms to restrain their activity. Although these elements can replicate within the genome, their mutation rates are rather limited. In contrast, ERVs are derived from exogenous retroviruses that can be horizontally transmitted between animals. Exogenous retroviruses can evolve much faster than EREs [141] and, in the case of a germ-line colonization, may represent new genomic elements that have few or no similarities to EREs already residing in the host genome. Thus, the host is forced to quickly develop an effective repression mechanism.

Germ-line colonization by ERV-like LTR retrotransposons has been described in insects [142]. However, vertebrates and tetrapods have accumulated a much more diverse retroviral load during evolution than other animals [64, 66]. What caused the immense ERV diversity in these animals? Many retroviruses that infect mammals replicate by co-opting properties of immune cells that normally operate during intercellular communication, such as antigen presentation and T-cell activation [143]. Thus, cells of the adaptive immune system, which first emerged in jawed vertebrates [144], might have served as replication sites for retroviruses and therefore favored ERV diversity. On the other hand, adaptive immunity might have exerted selective pressure on retroviruses, contributing to the rapid diversification of these parasites. Either way, it is possible that the KRAB-ZFP repression system evolved in response to the increasingly diverse burden of horizontally transmittable retroviruses in tetrapods.

**Fig. 2:**
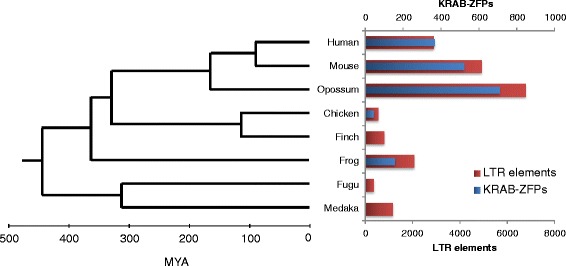
An evolutionary arms race between EREs and KRAB-ZFPs. Estimated number of LTR elements [64] and KRAB-ZFPs [15] in vertebrates. The phylogenetic tree is an approximate reprint of a previously published tree [64]

To our knowledge, only human and murine KRAB-ZFPs have been analyzed in genome-wide binding studies so far. One can therefore only speculate that EREs are indeed the main drivers of KRAB-ZFP diversification in other mammals and non-mammalian tetrapods. Consistent with this idea, a computational analysis of 16 mammalian genomes revealed a striking correlation between the number of endogenous LTR elements and the number of tandem-ZFP genes (Fig. 2) [64]. Moreover, the emergence of new LTR elements correlated with tandem-ZFP gene duplication events [64]. Surprisingly, the same correlation was observed in a selection of nonmammalian vertebrates, in which only few or none of the tandem-ZFPs contain KRAB domains [64]. It was therefore speculated that these species use tandem-ZFPs with alternative repressor domains to restrict EREs [64]. Indeed, the BTB/POZ domain, found in both vertebrate and invertebrate tandem-ZFPs, interacts with HDAC co-repressor complexes [145].

Some KRAB-ZFPs may even restrict retroviral activity without the help of transcriptional corepressors. The KRAB-ZFP associated SCAN domain is not only believed to be derived from a retrotransposon but is also structurally similar to the HIV C-terminal capsid [18]. Furthermore, the ability of the SCAN domain to multimerize by a domain-swapping mechanism resembles the multimerization of capsid domains to form the capsid structure of retroviruses [146, 147]. It was therefore speculated that this domain may target KRAB-ZFPs to cytoplasmic retroviral capsids, allowing sequestration of newly synthesized retroviral DNA [18]. Thus, it is imaginable that the exaptation of the SCAN domain enabled KRAB-ZFPs to restrict retroviral activity in nonmammalian tetrapods, possibly before the KRAB domain was able to interact with KAP1. The emergence of KAP1-interacting KRAB domains may then have provided an additional repression mechanism. Importantly, KRAB/KAP1 transcriptionally represses both chromosomal and nonintegrated DNA [148] and KAP1 inhibits genomic integration of HIV [149], suggesting that KAP1 can restrict retroviral replication by multiple mechanisms. Intriguingly, several tandem-ZFPs with a SCAN domain in lizards were predicted to bind Gmr1-like EREs, one of them precisely at the PBS [18]. One may therefore speculate that the SCAN domain played an important role in the evolution of ERE repressing KRAB-ZFPs. However, experimental evidence for retrotransposon repression by the SCAN domain or non-mammalian KRAB-ZFPs in general is still lacking.

The hypothesis that KRAB-ZFPs evolved to defend host genomes from parasitic DNA implies that many of these transcription factors bind to ERVs and other EREs. Using computational motif prediction tools, it was predicted that many human KRAB-ZFPs bind EREs that entered the human genome around the time these KRAB-ZFPs appeared [150]. Recently, about 70 randomly selected human ZFPs were epitope tagged and expressed in a human cell line to identify their genome-wide binding patterns by ChIP-seq. Of 18 KRAB-ZFPs, 16 bound to some extent to specific EREs, versus only about 10 % of non-KRAB ZFPs [9]. Thus, the majority of human KRAB-ZFPs can bind EREs, although it is not known whether they are required for ERE repression. Intriguingly, the estimated ages of most ERE-binding KRAB-ZFPs correlate with the ages of the EREs they bind [9]. However, two KRAB-ZFPs that are well conserved in mammals, ZNF382 and ZNF33A, bind to currently active LINE L1HS-like elements and hominoid-specific SVA elements, respectively [9]. Thus, these EREs are not likely the primary targets but they simply tolerate KRAB-ZFP binding, or alternatively, these KRAB-ZFPs were recently co-opted to repress EREs. In support of the latter possibility, there is a strong signature of recent positive selection at the ZNF33A locus in humans [9].

Evidence for an ongoing evolutionary arms race between host KRAB-ZFPs and transposable elements also came from the few studies that identified individual ERE-repressing KRAB-ZFPs. Both Gm6871 and KAP1 bind predominantly to L1 elements that entered the mouse genome 4–7 million years ago [10]. Similarly, human KAP1 binds preferentially to L1 elements estimated to be 8–27 million years old [10]. Moreover, in human ESCs, the youngest human L1 elements that are not bound by KAP1 are expressed at higher levels than their older counterparts. Depletion of the three DNA methyltransferases strongly upregulated these young L1 elements, but older L1 families were relatively unaffected [10]. The PIWI-piRNA pathway is involved in L1 silencing in human pluripotent stem cells [106]. In a proposed model, expression of newly emerging L1 elements is silenced by the PIWI-piRNA system, which is targeted to these elements by L1-derived piRNAs. Over time, KRAB-ZFPs evolve to recognize those transposable elements and take over repression until their target EREs become too degenerated to be recognized. By that time, the accumulated mutations and deletions of the EREs had already led to their inactivation so repression is no longer required [10]. Indeed, vertebrate genomes have many tandem-ZFP pseudogenes [64], suggesting that many KRAB-ZFP genes have become obsolete after their target EREs had been inactivated by genetic drift.

ZNF91 and ZNF93 emerged in the last common ancestor of apes and Old-World monkeys and are members of a KRAB-ZFP cluster that has amplified and diversified throughout the evolution of apes and humans [49]. Both ZNF91 and ZNF93 underwent several drastic structural changes in the last common ancestor of orangutans and humans 12–18 million years ago [8]. Intriguingly, these changes were crucial for the ability of ZNF91/93 to repress retrotransposons in humans. Around the same time as the ZNF91/93 changes, the ZNF93-targeted L1 elements also changed substantially. A 129-bp sequence within L1PA subgroups is lost in evolutionarily younger L1PA elements, indicating a potential repression escape mutation that allowed these elements to be expressed [8].

**Fig. 3:**
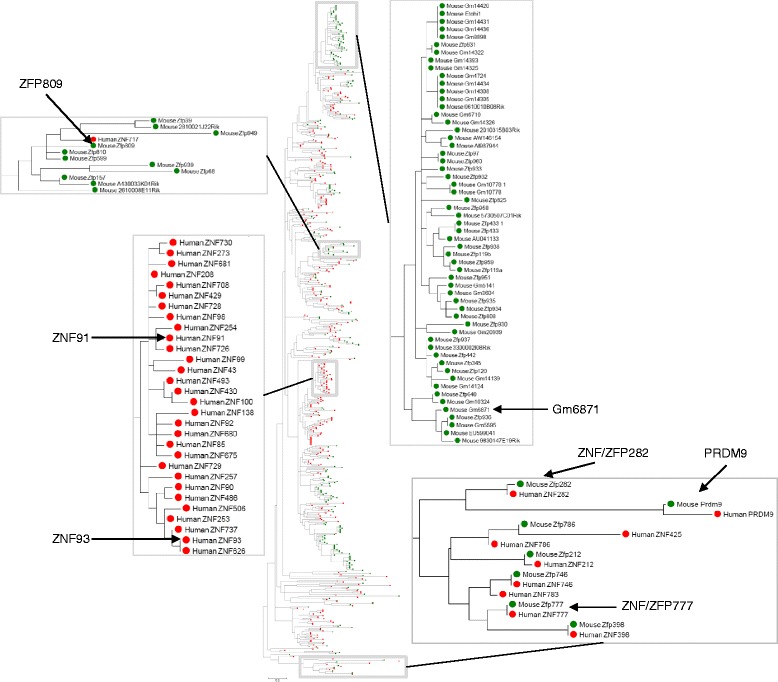
Phylogenetic tree of mouse and human KRAB-ZFPs. 277 mouse (green circles) and 339 human (red circles) KRAB-ZFP sequences (all proteins with both KRAB and C2H2 zinc finger domains) were retrieved from the UCSC Gene Sorter tool (https://genome.ucsc.edu/). KRAB domains were annotated through a Pfam domain (PF01352) screen (http://pfam.xfam.org/), extracted, and aligned with MUSCLE [197] to infer a Maximum-likelihood phylogenetic tree using MEGA version 6 with default parameters [198]. All KRAB sequences are provided as Additional file 1. Exemplary proportions of the tree that contain ERE-silencing KRAB-ZFPs or KRAB-ZFPs conserved between mouse and human (e.g., PRDM9, ZNF282, and ZNF777) are shown in more detail

Altogether these findings imply that evolutionarily young KRAB-ZFPs repress retroviruses and EREs, whereas older and well-conserved KRAB-ZFPs fulfill other functions. To gain insight into the evolutionary history of ERE repressing KRAB-ZFPs, we generated a phylogenetic tree of murine and human KRAB-ZFPs. Indeed, ZFP809 and Gm6871 are closely related to other murine KRAB-ZFPs but do not phylogenetically cluster with any human KRAB-ZFPs. On the other hand, the L1 and SVA repressors ZNF91/93 form a cluster with many other human KRAB-ZFPs but are not closely related to any mouse KRAB-ZFP (Fig. 3). In contrast, ancient and well-conserved KRAB-ZFPs such as PRDM9, ZFP/ZNF282 and ZNF/ZFP777 are present as one-to-one orthologues in mice and humans and do not group in clusters of species-specific KRAB-ZFPs (Fig. 3). This indicates that some KRAB-ZFPs are constrained in mammalian evolution whereas others, including the ancestors of ERE repressors such as ZFP809 and ZNF91/93, are prone to frequent gene duplications and diversification. This is in agreement with the model of KRAB-ZFP amplification and diversification as a response to invading foreign DNA.

**Fig. 4:**
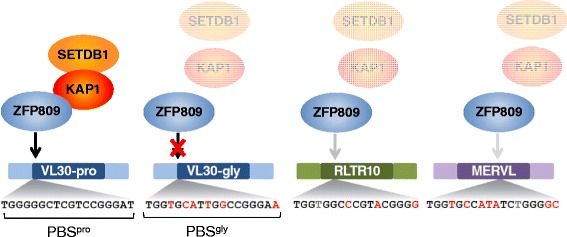
Differential ZFP809 binding to various ERVs. ZFP809 target sequences identified by ChIP-seq [7] are shown with differences from the canonical PBS^pro^ highlighted in red. Dashed arrows indicate weak ZFP809 binding that is not sufficient to form the KAP1/SETDB1 repressor complex

ZFP809 binds to VL30 and MmERV elements (both ERVs of the ERV1 family) that contain a PBS^pro^, but many of these elements contain a PBS complementary to a glycine tRNA instead (PBS^gly^) [7] (Fig. 4). Thus, even closely related elements within the same ERV group can escape repression by a specific KRAB-ZFP. At the same time, ZFP809 binds weakly to several hundred genomic RLTR10 and MERVL elements, ERVs belonging to the ERVK and ERVL families, respectively (Wolf et al., *unpublished data*). Although target motifs similar to the PBS^pro^ can be found at these ZFP809 binding sites (Fig. 4), no co-occupation with KAP1 and SETDB1 was observed, presumably because ZFP809 binding is not sufficient to assemble the KAP1 repressor complex at these targets ([7] and Wolf et al. *unpublished data*). However, the weak binding affinity of ZFP809 to these elements also shows that imperfect binding sites for an ERV-repressing KRAB-ZFP can appear by chance in unrelated ERVs, possibly because of the general GC richness in these elements. ERV-targeting KRAB-ZFPs might have a general potential to bind weakly to other ERVs. If such a KRAB-ZFP is duplicated or becomes available after its original target ERV was inactivated over time, mutations of the ZNFs might allow stronger binding and therefore functional silencing of newly emerged ERVs.

### A possible link between KRAB-ZFPs and ERE adaptation

#### Transcriptional regulation of genes through ERE repressing KRAB-ZFPs

EREs and especially ERVs have a profound impact on patterns of mammalian gene expression. Retroviral LTRs contain strong promoter elements to ensure efficient expression of their proviral genome. When integrated near a cellular gene, transcription from these LTRs can drive the expression of that gene [151, 152]. Furthermore enhancers within LTRs can influence the expression of distant cellular genes and contribute to the innovation of gene regulatory networks [68, 69, 153–156].

**Fig. 5:**
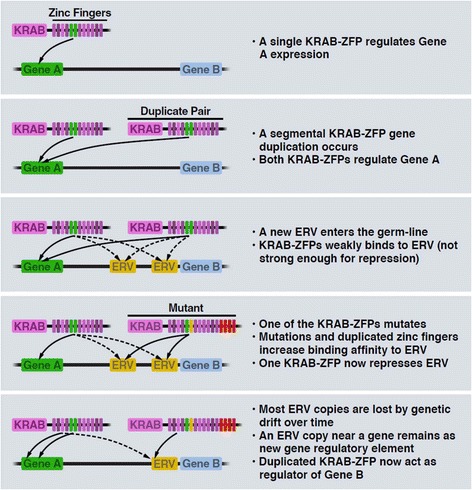
Hypothetical model of ERV/KRAB-ZFP adaption as regulators of gene expression. See Figure for explanations

As discussed above, KRAB-ZFPs that repress newly emerged EREs may result from a duplication of an existing ERE repressor, followed by mutations in the DNA binding domain that leads to recognition of the new targets. Alternatively, gene-targeting KRAB-ZFPs might duplicate and change their binding specificity towards new EREs (Fig. 5). Furthermore, since EREs have been continually co-opted as gene regulatory elements, it is plausible that some KRAB-ZFPs regulate gene expression by binding to EREs (Fig. 5). Indeed, knockout of KAP1 not only de-repressed ERVs but also many genes near those ERVs [157]. Also ZFP809 knockout led to the upregulation of a handful of cellular genes near ZFP809-targeted ERVs [7].

It has been also reported that an IAP LTR that drives gene expression is silenced by a gene located in a KRAB-ZFP cluster [158]. Although this might be another example of an ERV/KRAB-ZFP that got co-opted as a gene regulator, the identity of the repressor gene is yet to be determined. Furthermore, a human-specific SVA element that integrated into the fibroblast growth factor 2 (FGF2) promoter (Greenberg et al. *unpublished data*) may enhance the expression of this key protein during brain development [159], possibly regulated by ZNF91 [8].

#### ZFP57 protects genomic imprints in retrotransposed genes

One of the best-characterized KRAB-ZFPs, ZFP57, is required to maintain a subset of genomic imprints in mice [160], and mutations in human ZFP57 have been associated with transient neonatal diabetes [161]. ZFP57 binds to a methylated hexanucleotide within imprinted control regions and recruits KAP1 and SETDB1 to establish H3K9me3 [29, 162]. By binding to the methylated imprinted control region, ZFP57 also protects the methylated region from the genome-wide demethylation that occurs during mammalian preimplantation development. Although the imprinted control regions bound by ZFP57 in mammals are not associated with particular EREs, five murine imprinted genes arose by retrotransposition (Mcts2, Nap1l15, U2af1-rs1, Inpp5f_v2, and Peg12), and another two are derived from retrotransposons (Rtl1 and Peg10) [163]. Furthermore, DNA methylation is considered to have evolved primarily as a defense against foreign DNA [95]. Thus, genomic imprinting itself and its maintenance by KRAB-ZFPs might originate from retrotransposon repression.

#### KRAB-ZFPs regulate sexually dimorphic gene expression patterns through ERV targeting

Sexually dimorphic gene expression in liver is a complex phenomenon in mice and humans. Sex-specific expression of growth hormones can induce gene expression, and genes can be repressed in a sex-specific manner. The mouse regulator of sex-limitation (*Rsl*) locus encodes two KRAB-ZFP genes, *Rsl1* and *Rsl2*, which are regulated directly in the kidney by androgen or indirectly in the liver by growth hormones [164].

One *Rsl*-repressed gene, *Cyp2d9*, is a member of the large cytochrome P450 family, which participates in many metabolic processes, such as detoxification of foreign chemicals, hormone synthesis and breakdown, and cholesterol synthesis [165]. *Cyp2d9* and other sexually dimorphic cytochrome P450 genes were also upregulated in KAP1 knockout liver [166], confirming a role for KRAB-ZFPs in sexually dimorphic patterns of gene expression. Interestingly, cytochrome P450 genes have been diversified in mammals through gene duplications and positive selection, similar to KRAB-ZFP genes [167]. Because of their abundance and evolutionary dynamic, these genes might be prone to ERV-mediated repression by KRAB-ZFPs. Indeed, one of the few genes that was upregulated in ZFP809 knockout mice was a cytochrome P450 gene (*Cyp4f37*) that contained an ancient ERV insertion near the promoter region [7].

More importantly, another target of *Rsl*, which encodes sex-limited protein (Slp) was reported to be controlled by an ancient ERV LTR located 2 kb upstream of this gene [168, 169]. Indeed, Rsl1 binds a defined sequence within this LTR, suggesting that Rsl1-mediated control of Slp evolved from retroviral repression [170]. Intriguingly, *Rsl1* is located within a cluster of recently duplicated KRAB-ZFP genes found only in the *Mus* lineage [171].

#### From meiotic recombination control to ERV-repression

In most mammals, homologous recombination during meiosis tends to occur at specific segments of the genome. Interestingly, the placement and activity of these so-called hotspots varies greatly between closely related *Mus* species [172], between humans and primates [173, 174], and even between human individuals [175]. These hotspots are enriched in H3K4me3, a histone mark usually found at active and poised enhancers. Human recombination hotspots often contain a 13-mer sequence motif [176]. The KRAB-ZFP PRDM9 governs recombination activity in humans and mice [177–179] through the H3K4 trimethyltransferase activity of its SET domain [13, 14]. Intriguingly, many human PRDM9 alleles have been identified [180], and accelerated evolution of the PRDM9 DNA binding domain has been reported [181–183]. PRDM9 was therefore suggested to bind rapidly evolving repetitive DNA elements [176, 181]. Indeed, THE1A and THE1B LTR elements, members of the Mammalian apparent LTR-retrotransposons (MaLRs) family, contain a PRDM9 binding motif and are overrepresented in PRDM9-associated hotspots [183, 184].

Could this indicate that PRDM9 originally evolved as an ERV repressor? Although PRDM9 predates the emergence of human PRDM9-bound THE1 LTR elements, MaLRs colonized the genomes of eutherian mammals at least 80–100 million years ago [185]. An ancient connection between LTR elements and PRDM9 in mammals can therefore not be excluded. However, the KRAB domain of PRDM9 lacks the amino acid sequences that have been identified as essential for KAP1 interaction [15, 31], indicating that PRDM9 is not a part of the KRAB/KAP1 ERV repression system. Nevertheless, ERV integrations might have re-organized recombination hotspots by introducing new PRDM9 binding sites. Moreover, the mechanism of DNA binding by PRDM9—which is believed to be highly specific yet permissive at the same time [176, 186]—and its ability to rapidly change DNA specificity may have been the perfect attributes to trigger expansion and evolution of ERV-repressing KRAB-ZFPs.

### Challenges and future directions of KRAB-ZFP research

Despite recent progress, KRAB-ZFPs are not only one of the largest but also one of the least understood transcription factor families in mammals. In fact, many functional KRAB-ZFP genes might not even be annotated yet, whereas some predicted KRAB-ZFPs will turn out to be pseudogenes. The highly repetitive nature of KRAB-ZFP genes makes conventional annotations difficult, and estimates of their copy numbers vary as they strongly depend on the inclusion criteria used [1, 2, 6, 15]. Especially in low-quality genomes, the real number might be underestimated, and alternative splice isoforms may additionally contribute to the diversity of expressed KRAB-ZFPs.

Moreover, the assumption that all KRAB-ZFPs are DNA binding transcription factors might be premature and it is possible that some tandem-ZFPs function outside the nucleus. Binding of C2H2-type ZNFs to RNA and proteins has been reported [187, 188], suggesting that some KRAB-ZFPs do not act as DNA binding transcription factors. Cross-linking and immunoprecipitation followed by RNA sequencing (CLIP-seq) analysis of”orphan” C2H2-type ZNFs not known to bind DNA or for characterized ZNFs that contain orphan ZNFs might yield novel insights into RNA biology and RNA recognition.

**Fig. 6:**
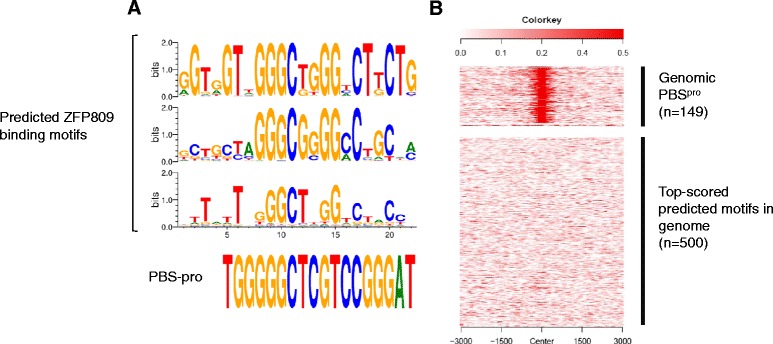
Comparison of predicted ZFP809 target motifs and experimentally identified target sequence of ZFP809. **a** The ZFP809 protein sequence was used to infer predicted target motifs using an expanded linear support vector machine (SVM) (top) or a polynominal SVM (middle) [27] and a prediction tool based on bacterial one-hybrid screens and ChIP-seq data (bottom) [9]. The canonical PBS^pro^ sequence is shown below. **b** One of the predicted motifs [9] was used to screen matching genomic sites of the mouse genome using the software tool FIMO [199]. The 500 top-scored sites and the 149 genomic PBS^pro^ were screened for ZFP809 enrichment by NGS.plot [200] and published FLAG-ZFP809 ChIP-seq data in murine embryonic carcinoma cells [7]

A key to understanding the KRAB-ZFPs that function as DNA binding transcription factors is to determine their genome-wide binding patterns. Although ZNF prediction tools are improving, they cannot, and perhaps never will, be used to reliably predict genome-wide DNA binding sites. We used several of these tools to predict a ZFP809 binding motif and compared the outcome with the experimentally determined ZFP809 binding site, the PBS^pro^ [7, 112]. Although the predicted motifs showed some similarity to the PBS^pro^, the fraction of overlapping nucleotides was rather small (Fig. 6a). ZFP809 and probably most KRAB-ZFPs tolerate very few mismatches for efficient binding [112]. Therefore, every single falsely predicted nucleotide drastically increases the proportion of falsely predicted binding sites in the genome. To test the accuracy of the predicted ZFP809 binding motif, we screened the mouse genome for targets resembling this motif and analyzed the 500 top-scored genomic sites for ZFP809 enrichment using published ChIP-seq data [7]. Indeed, ZFP809 was not enriched at these predicted genomic binding sites (Fig. 6b). Although the predicted motifs of some KRAB-ZFPs strikingly resemble the experimentally determined target motif [9], the case of ZFP809 highlights that KRAB-ZFP binding prediction without experimental testing remains highly unreliable.

Improved ChIP-seq protocols such as ChIP-exo and ChIP-nexus allow transcription factor binding sites to be determined at near nucleotide resolution [189, 190]. These new techniques and the reduced cost of high-throughput sequencing applications will greatly facilitate identification of the genomic binding patterns of mammalian KRAB-ZFPs within the next few years. Importantly, these data will also help to improve tandem-ZFP prediction tools. However, the similarity between KRAB-ZFPs makes the generation of specific antibodies extremely challenging. Overexpression of epitope-tagged KRAB-ZFPs might partially overcome this problem [7, 9]. However, this approach can be misleading because overexpression of KRAB-ZFPs can lead to the identification of binding sites that would not be bound by the protein expressed at normal levels [7]. It is also unknown whether ChIP-seq with overexpressed KRAB-ZFPs in a certain cell type will indicate the binding sites in other cells or tissues, since possibly cell-specific posttranslational modifications can influence the DNA binding properties of KRAB-ZFPs [191].

KRAB-ZFPs have evolved through natural section to bind precisely to sequences that needed to be transcriptionally repressed (e.g. ERVs). Nevertheless, binding to imperfect target sites is likely to occur. We speculate that such binding is kept to a minimum by selectively expressing KRAB-ZFPs at relatively low levels, ensuring that only the preferred binding sites are occupied. However, it is possible that, by regulating KRAB-ZFP expression levels, different cell types can determine the number of functionally relevant KRAB-ZFP binding sites. A powerful strategy to circumvent KRAB-ZFP overexpression in genome-wide binding assays will be tagging of endogenous KRAB-ZFP genes with epitopes using the CRISPR/Cas9 system.

Ultimately, loss-of-function studies will be required to determine the functional roles of KRAB-ZFPs. However, the repetitive and clustered characteristics of KRAB-ZFP genes—especially of evolutionarily young ones that have recently duplicated—remain a major obstacle. Conventional gene targeting by homologous recombination as well as RNAi- and CRISPR/Cas9-based approaches depend on unique sequences to ensure that only the right target is affected by knockdown or knockout. Alternatively, gene-trap collections of ESC clones might be used to generate KRAB-ZFPs knockout mice. However, mapping of gene-trap insertions at repetitive genes is problematic as well.

Thus new strategies will be needed to test the biological requirements of individual KRAB-ZFPs. We recommend targeting the rather unique regions between KRAB domains and ZNFs with CRISPR/Cas9 guide RNAs to mutate KRAB-ZFP genes. However, in the case of recently duplicated KRAB-ZFP genes, even these regions might be too similar for specific targeting by CRISPR guide-RNAS or siRNAs. Since recently duplicated KRAB-ZFP genes are usually located in close proximity, one might consider genetic depletion of several KRAB-ZFP genes within a cluster at once by inserting loxP sites in the unique flanking regions using CRISPR/Cas9 or conventional gene targeting, followed by Cre-mediated recombination. Stepwise rescue experiments and ChIP-seq with epitope-tagged proteins may be used to assign the observed phenotypes in such KRAB-ZFP cluster knockout cells or animals to a single gene.

Once a larger number of KRAB-ZFPs have been characterized in detail, several important questions about the function and evolution of KRAB-ZFPs can be addressed. For instance, little is known about the time it takes for KRAB-ZFPs to evolve to bind newly emerged sequences such as ERVs. It is also somewhat puzzling how KRAB-ZFPs can keep up with active and therefore mutating ERVs. One possibility is that KRAB-ZFPs (such as ZFP809) that bind to retroviral sequences that are at least partially conserved in various ERV groups are preferably selected as repressors of parasitic elements. Alternatively, KRAB-ZFPs might primarily repress ERVs and other retrotransposons that have already lost the ability to replicate, whereas other repression mechanisms act on newly emerged active ERE families, as previously suggested [10]. Nevertheless, insights could be gained by experimental testing how long (how many mutations) it takes for a KRAB-ZFP to start binding to a new ERE. One possibility would be to use a target that is only weakly bound by a KRAB-ZFP and test a large number of KRAB-ZFP mutants in a high-throughput screen to test how many mutations it takes to improve binding to the new target. Such an assay would also be useful to improve the target specificity of artificially engineered KRAB-ZFPs. In the long term, such optimized engineered KRAB-ZFPs might be used to suppress transposable elements and genes that cause disease in patients. Furthermore, replacing the KRAB domain with activating or other functional domains will allow us to tightly control expression of mobile DNA and regular genes. This approach might be used to induce transcription of ERE-linked genes to boost stem cell pluripotency or help differentiation into certain tissues.

The current KRAB-ZFP sets in mammals are likely a mixture of KRAB-ZFPs that are under purifying or positive selection and KRAB-ZFPs that arose by recent gene duplications and subsequent mutations but remain nonessential for the host. These KRAB-ZFPs will become pseudogenes and eventually disappear by genetic drift. Additionally, one might expect that duplicating and mutating KRAB-ZFPs sometimes reduce host fitness and are therefore rapidly removed by negative selection. How many useless or harmful KRAB-ZFPs are necessary before a beneficial one evolves? This question could be addressed by analyzing the genomes of very closely related species (e.g., wild mice and domestic mouse strains). One might even expect that there are differences in the number of KRAB-ZFPs between individuals of the same species. More importantly, germ-line mutations and segmental duplications of KRAB-ZFPs might influence human development or cause disorders and disease. Indeed, the KRAB-ZFP ZNF568 exist as three different alleles in humans, and a correlation between these alleles and the brain size of newborns was reported [192].

According to a recent study, natural occurring nonsynonymous single nucleotide polymorphisms (SNPs) at “specificity residues” of human tandem-ZFPs are rare, indicating that ZNF mutations that change the binding specificity of tandem-ZFPs are rapidly removed from the population by negative selection [193]. Furthermore, these rare SNPs generally do not correlate with altered gene expression profiles [193]. However, the highly repetitive nature of tandem-ZFP genes, especially at the ZNF coding regions, makes SNP calling extremely challenging and might have resulted in an underestimation of such polymorphisms. Moreover, conventional expression databases do not report expression levels of repetitive elements and SNP effects on ERE expression might therefore have remained undetected. Although a re-analysis of existing RNA-seq data might reveal ERE repression deficiencies associated with mutations in tandem-ZFP genes, many RNA-seq studies are still based on short (36 bp) read sequencing, which makes it problematic to assign reads to individual ERE copies and therefore hinders accurate quantification of ERE expression. Furthermore, the study excluded frameshift mutations and did not try to identify the loss or duplication of tandem-ZFPs in individuals [193]. Importantly, tandem-ZFP clusters on human chromosome 19 have been associated with unusually high copy number variation [150]. Although it will be difficult to identify events such as duplications of a single KRAB-ZFP by genome analysis, a thorough analysis of high coverage genome sequencing data and 100 bp paired-end RNA-seq data might lead to the identification of physiologically relevant KRAB-ZFP polymorphisms in humans.

Little is known about how a loss-of-function mutation of a single ERE-targeting KRAB-ZFP would affect the host organism. While reactivation of a replication-competent ERV or a high-copy retrotransposon could have immediate deleterious consequences for the host, de-repression of non-autonomous ERVs or low copy transposons might only have subtle effects. In support of this idea, the drastic upregulation of a small subset of non-autonomous VL30 elements in ZFP809 knockout mice did not seem to impair their health or fitness [7]. Nevertheless, these mice were not monitored for more than two generations; deleterious effects might have emerged in later generations. Another possibility is that potentially hazardous ZFP809-repressed ERVs are polymorphic among mouse strains and simply not present in the strain that was used in this study. Furthermore, ERV reactivation caused by KRAB-ZFP deletion might impair the host only under certain circumstances, such as physiological stress or during pathogen infection.

Interestingly, it seems that SVA and SVA-related LAVA elements have expanded in some primates that lack ZNF91 [8, 194–196], suggesting that ZNF91 prevents genomic SVA amplification. Moreover, polymorphic human KRAB-ZFPs may allow for mobilization of DNA in a certain haplogroup/population. Indeed, certain island populations carry SVA insertions [87] that may result from ZNF91 mutations. Future work on KRAB-ZFP knockout mouse models and genome-wide association studies of human KRAB-ZFP polymorphisms will reveal how a failure of the KRAB-ZFP ERE repression system impacts fitness and health of a host.

## Conclusions

It is increasingly evident that transposable elements have a profound impact on mammals. Therefore, understanding the factors that keep these elements under control is of high importance for both basic and applied medical research. The recent evidence summarized in this review strongly supports the hypothesis that a large fraction of KRAB-ZFPs evolved to bind and possibly repress mobile parasitic DNA in mammals. However, since only a very small number of KRAB-ZFPs have been thoroughly investigated, the true spectrum of KRAB-ZFP functions cannot be anticipated yet. Without doubt, future research will yield exciting and unexpected insights into this enigmatic protein family.

## Additional file

Additional file 1:
**KRAB domain sequences of human and mouse KRAB-ZFPs in Multi-FASTA format.** See Fig. 3 for details. (FA 34 kb)

## Abbreviations

ERE: Endogenous retroelement
ERV: Endogenous retrovirus
ESC: Embryonic stem cell
gpt: Glutamic-pyruvate transaminase
HDAC: Histone deacetylase
HERV: Human endogenous retrovirus
IAP: Intracisternal A-particle
KRAB-ZFP: Krüppel-associated box zinc finger protein
LINE: Long interspersed nuclear element
MuLV: Murine leukemia virus
piRNA: PIWI-interacting RNA
Rsl: Regulator of sex-limitation
SVA: SINE-VNTR-Alu, SVA
SINE: Short interspersed nuclear element
Slp: Sex-limited protein
SNP: Single nucleotide polymorphism
siRNA: Short interfering RNA
ZNF: Zinc finger

## References

[1] Emerson RO, Thomas JH (2009). Adaptive evolution in zinc finger transcription factors. PLoS Genet.

[2] Urrutia R (2003). KRAB-containing zinc-finger repressor proteins. Genome Biol.

[3] Hauschild-Quintern J, Petersen B, Cost GJ, Niemann H (2013). Gene knockout and knockin by zinc-finger nucleases: current status and perspectives. CMLS.

[4] Mandell JG, Barbas CF (2006). Zinc Finger Tools: custom DNA-binding domains for transcription factors and nucleases. Nucleic Acids Res.

[5] Lupo A, Cesaro E, Montano G, Zurlo D, Izzo P, Costanzo P (2013). KRAB-Zinc Finger Proteins: A Repressor Family Displaying Multiple Biological Functions. Current genomics.

[6] Huntley S, Baggott DM, Hamilton AT, Tran-Gyamfi M, Yang S, Kim J (2006). A comprehensive catalog of human KRAB-associated zinc finger genes: insights into the evolutionary history of a large family of transcriptional repressors. Genome Res.

[7] Wolf G, Yang P, Fuchtbauer AC, Fuchtbauer EM, Silva AM, Park C (2015). The KRAB zinc finger protein ZFP809 is required to initiate epigenetic silencing of endogenous retroviruses. Genes Dev.

[8] Jacobs FM, Greenberg D, Nguyen N, Haeussler M, Ewing AD, Katzman S (2014). An evolutionary arms race between KRAB zinc-finger genes ZNF91/93 and SVA/L1 retrotransposons. Nature.

[9] Najafabadi HS, Mnaimneh S, Schmitges FW, Garton M, Lam KN, Yang A (2015). C2H2 zinc finger proteins greatly expand the human regulatory lexicon. Nat Biotechnol.

[10] Castro-Diaz N, Ecco G, Coluccio A, Kapopoulou A, Yazdanpanah B, Friedli M (2014). Evolutionally dynamic L1 regulation in embryonic stem cells. Genes Dev.

[11] Turelli P, Castro-Diaz N, Marzetta F, Kapopoulou A, Raclot C, Duc J (2014). Interplay of TRIM28 and DNA methylation in controlling human endogenous retroelements. Genome Res.

[12] Birtle Z, Ponting CP (2006). Meisetz and the birth of the KRAB motif. Bioinformatics.

[13] Mihola O, Trachtulec Z, Vlcek C, Schimenti JC, Forejt J (2009). A mouse speciation gene encodes a meiotic histone H3 methyltransferase. Science.

[14] Kono H, Tamura M, Osada N, Suzuki H, Abe K, Moriwaki K (2014). Prdm9 polymorphism unveils mouse evolutionary tracks. DNA Res.

[15] Liu H, Chang LH, Sun Y, Lu X, Stubbs L (2014). Deep vertebrate roots for mammalian zinc finger transcription factor subfamilies. Genome Biol Evol.

[16] Mouse Genome Sequencing C, Waterston RH, Lindblad-Toh K, Birney E, Rogers J, Abril JF (2002). Initial sequencing and comparative analysis of the mouse genome. Nature.

[17] Meroni G (2012). Genomics and evolution of the TRIM gene family. Adv Exp Med Biol.

[18] Emerson RO, Thomas JH (2011). Gypsy and the birth of the SCAN domain. J Virol.

[19] Brayer KJ, Segal DJ (2008). Keep your fingers off my DNA: protein-protein interactions mediated by C2H2 zinc finger domains. Cell Biochem Biophys.

[20] Hoffmann A, Ciani E, Boeckardt J, Holsboer F, Journot L, Spengler D (2003). Transcriptional activities of the zinc finger protein Zac are differentially controlled by DNA binding. Mol Cell Biol.

[21] Hata A, Seoane J, Lagna G, Montalvo E, Hemmati-Brivanlou A, Massague J (2000). OAZ uses distinct DNA- and protein-binding zinc fingers in separate BMP-Smad and Olf signaling pathways. Cell.

[22] Johnson DS, Mortazavi A, Myers RM, Wold B (2007). Genome-wide mapping of in vivo protein-DNA interactions. Science.

[23] Pavletich NP, Pabo CO (1993). Crystal structure of a five-finger GLI-DNA complex: new perspectives on zinc fingers. Science.

[24] Nakahashi H, Kwon KR, Resch W, Vian L, Dose M, Stavreva D (2013). A genome-wide map of CTCF multivalency redefines the CTCF code. Cell reports.

[25] Pavletich NP, Pabo CO (1991). Zinc finger-DNA recognition: crystal structure of a Zif268-DNA complex at 2.1 A. Science.

[26] Gupta A, Christensen RG, Bell HA, Goodwin M, Patel RY, Pandey M (2014). An improved predictive recognition model for Cys(2)-His(2) zinc finger proteins. Nucleic Acids Res.

[27] Persikov AV, Singh M (2014). De novo prediction of DNA-binding specificities for Cys2His2 zinc finger proteins. Nucleic Acids Res.

[28] Liu Y, Zhang X, Blumenthal RM, Cheng X (2013). A common mode of recognition for methylated CpG. Trends Biochem Sci.

[29] Quenneville S, Verde G, Corsinotti A, Kapopoulou A, Jakobsson J, Offner S (2011). In embryonic stem cells, ZFP57/KAP1 recognize a methylated hexanucleotide to affect chromatin and DNA methylation of imprinting control regions. Mol Cell.

[30] Friedman JR, Fredericks WJ, Jensen DE, Speicher DW, Huang XP, Neilson EG (1996). KAP-1, a novel corepressor for the highly conserved KRAB repression domain. Genes Dev.

[31] Margolin JF, Friedman JR, Meyer WK, Vissing H, Thiesen HJ, Rauscher FJ (1994). Kruppel-associated boxes are potent transcriptional repression domains. Proc Natl Acad Sci U S A.

[32] Peng H, Begg GE, Schultz DC, Friedman JR, Jensen DE, Speicher DW (2000). Reconstitution of the KRAB-KAP-1 repressor complex: a model system for defining the molecular anatomy of RING-B box-coiled-coil domain-mediated protein-protein interactions. J Mol Biol.

[33] Schultz DC, Friedman JR, Rauscher FJ (2001). Targeting histone deacetylase complexes via KRAB-zinc finger proteins: the PHD and bromodomains of KAP-1 form a cooperative unit that recruits a novel isoform of the Mi-2alpha subunit of NuRD. Genes Dev.

[34] Schultz DC, Ayyanathan K, Negorev D, Maul GG, Rauscher FJ (2002). SETDB1: a novel KAP-1-associated histone H3, lysine 9-specific methyltransferase that contributes to HP1-mediated silencing of euchromatic genes by KRAB zinc-finger proteins. Genes Dev.

[35] Wiznerowicz M, Jakobsson J, Szulc J, Liao S, Quazzola A, Beermann F (2007). The Kruppel-associated box repressor domain can trigger de novo promoter methylation during mouse early embryogenesis. J Biol Chem.

[36] Rowe HM, Friedli M, Offner S, Verp S, Mesnard D, Marquis J (2013). De novo DNA methylation of endogenous retroviruses is shaped by KRAB-ZFPs/KAP1 and ESET. Development.

[37] Lechner MS, Begg GE, Speicher DW, Rauscher FJ (2000). Molecular determinants for targeting heterochromatin protein 1-mediated gene silencing: direct chromoshadow domain-KAP-1 corepressor interaction is essential. Mol Cell Biol.

[38] Fuks F, Hurd PJ, Deplus R, Kouzarides T (2003). The DNA methyltransferases associate with HP1 and the SUV39H1 histone methyltransferase. Nucleic Acids Res.

[39] Groner AC, Meylan S, Ciuffi A, Zangger N, Ambrosini G, Denervaud N (2010). KRAB-zinc finger proteins and KAP1 can mediate long-range transcriptional repression through heterochromatin spreading. PLoS Genet.

[40] Thompson PJ, Dulberg V, Moon KM, Foster LJ, Chen C, Karimi MM (2015). hnRNP K coordinates transcriptional silencing by SETDB1 in embryonic stem cells. PLoS Genet.

[41] Ishiuchi T, Enriquez-Gasca R, Mizutani E, Boskovic A, Ziegler-Birling C, Rodriguez-Terrones D (2015). Early embryonic-like cells are induced by downregulating replication-dependent chromatin assembly. Nat Struct Mol Biol.

[42] Sadic D, Schmidt K, Groh S, Kondofersky I, Ellwart J, Fuchs C et al. Atrx promotes heterochromatin formation at retrotransposons. EMBO reports. 2015. doi:10.15252/embr.201439937. 10.15252/embr.201439937 PMC4515123 26012739

[43] Tchasovnikarova IA, Timms RT, Matheson NJ, Wals K, Antrobus R, Gottgens B (2015). Epigenetic silencing by the HUSH complex mediates position-effect variegation in human cells. Science.

[44] Rao SS, Huntley MH, Durand NC, Stamenova EK, Bochkov ID, Robinson JT (2014). A 3D map of the human genome at kilobase resolution reveals principles of chromatin looping. Cell.

[45] Vogel MJ, Guelen L, de Wit E, Peric-Hupkes D, Loden M, Talhout W (2006). Human heterochromatin proteins form large domains containing KRAB-ZNF genes. Genome Res.

[46] Hahn MA, Wu X, Li AX, Hahn T, Pfeifer GP (2011). Relationship between gene body DNA methylation and intragenic H3K9me3 and H3K36me3 chromatin marks. PLoS One.

[47] Frietze S, O’Geen H, Blahnik KR, Jin VX, Farnham PJ (2010). ZNF274 recruits the histone methyltransferase SETDB1 to the 3’ ends of ZNF genes. PLoS One.

[48] O’Geen H, Squazzo SL, Iyengar S, Blahnik K, Rinn JL, Chang HY (2007). Genome-wide analysis of KAP1 binding suggests autoregulation of KRAB-ZNFs. PLoS Genet.

[49] Hamilton AT, Huntley S, Tran-Gyamfi M, Baggott DM, Gordon L, Stubbs L (2006). Evolutionary expansion and divergence in the ZNF91 subfamily of primate-specific zinc finger genes. Genome Res.

[50] Schmidt D, Durrett R (2004). Adaptive evolution drives the diversification of zinc-finger binding domains. Mol Biol Evol.

[51] Nowick K, Hamilton AT, Zhang H, Stubbs L (2010). Rapid sequence and expression divergence suggest selection for novel function in primate-specific KRAB-ZNF genes. Mol Biol Evol.

[52] Bellefroid EJ, Poncelet DA, Lecocq PJ, Revelant O, Martial JA (1991). The evolutionarily conserved Kruppel-associated box domain defines a subfamily of eukaryotic multifingered proteins. Proc Natl Acad Sci U S A.

[53] Okumura K, Sakaguchi G, Naito K, Tamura T, Igarashi H (1997). HUB1, a novel Kruppel type zinc finger protein, represses the human T cell leukemia virus type I long terminal repeat-mediated expression. Nucleic Acids Res.

[54] Conroy AT, Sharma M, Holtz AE, Wu C, Sun Z, Weigel RJ (2002). A novel zinc finger transcription factor with two isoforms that are differentially repressed by estrogen receptor-alpha. J Biol Chem.

[55] Chung HR, Lohr U, Jackle H (2007). Lineage-specific expansion of the zinc finger associated domain ZAD. Mol Biol Evol.

[56] Nietfeld W, Conrad S, van Wijk I, Giltay R, Bouwmeester T, Knochel W (1993). Evidence for a clustered genomic organization of FAX-zinc finger protein encoding transcription units in Xenopus laevis. J Mol Biol.

[57] Kleefstra T, Yntema HG, Oudakker AR, Banning MJ, Kalscheuer VM, Chelly J (2004). Zinc finger 81 (ZNF81) mutations associated with X-linked mental retardation. J Med Genet.

[58] Kalsoom UE, Klopocki E, Wasif N, Tariq M, Khan S, Hecht J (2013). Whole exome sequencing identified a novel zinc-finger gene ZNF141 associated with autosomal recessive postaxial polydactyly type A. J Med Genet.

[59] Frietze S, Lan X, Jin VX, Farnham PJ (2010). Genomic targets of the KRAB and SCAN domain-containing zinc finger protein 263. J Biol Chem.

[60] Corsinotti A, Kapopoulou A, Gubelmann C, Imbeault M, de Sio FR S, Rowe HM (2013). Global and stage specific patterns of Kruppel-associated-box zinc finger protein gene expression in murine early embryonic cells. PLoS One.

[61] Barde I, Rauwel B, Marin-Florez RM, Corsinotti A, Laurenti E, Verp S (2013). A KRAB/KAP1-miRNA cascade regulates erythropoiesis through stage-specific control of mitophagy. Science.

[62] Santoni de Sio FR (2014). Kruppel-associated box (KRAB) proteins in the adaptive immune system. Nucleus.

[63] Nowick K, Gernat T, Almaas E, Stubbs L (2009). Differences in human and chimpanzee gene expression patterns define an evolving network of transcription factors in brain. Proc Natl Acad Sci U S A.

[64] Thomas JH, Schneider S (2011). Coevolution of retroelements and tandem zinc finger genes. Genome Res.

[65] Lander ES, Linton LM, Birren B, Nusbaum C, Zody MC, Baldwin J (2001). Initial sequencing and analysis of the human genome. Nature.

[66] Feschotte C, Gilbert C (2012). Endogenous viruses: insights into viral evolution and impact on host biology. Nat Rev Genet.

[67] Gifford WD, Pfaff SL, Macfarlan TS (2013). Transposable elements as genetic regulatory substrates in early development. Trends Cell Biol.

[68] Macfarlan TS, Gifford WD, Driscoll S, Lettieri K, Rowe HM, Bonanomi D (2012). Embryonic stem cell potency fluctuates with endogenous retrovirus activity. Nature.

[69] Chuong EB, Rumi MA, Soares MJ, Baker JC (2013). Endogenous retroviruses function as species-specific enhancer elements in the placenta. Nat Genet.

[70] Grow EJ, Flynn RA, Chavez SL, Bayless NL, Wossidlo M, Wesche DJ (2015). Intrinsic retroviral reactivation in human preimplantation embryos and pluripotent cells. Nature.

[71] Ohnuki M, Tanabe K, Sutou K, Teramoto I, Sawamura Y, Narita M (2014). Dynamic regulation of human endogenous retroviruses mediates factor-induced reprogramming and differentiation potential. Proc Natl Acad Sci U S A.

[72] Lowe CB, Bejerano G, Haussler D (2007). Thousands of human mobile element fragments undergo strong purifying selection near developmental genes. Proc Natl Acad Sci U S A.

[73] Conley AB, Piriyapongsa J, Jordan IK (2008). Retroviral promoters in the human genome. Bioinformatics.

[74] Lavialle C, Cornelis G, Dupressoir A, Esnault C, Heidmann O, Vernochet C (2013). Paleovirology of ‘syncytins’, retroviral env genes exapted for a role in placentation. Philos Trans R Soc Lond Ser B Biol Sci.

[75] Maksakova IA, Romanish MT, Gagnier L, Dunn CA, van de Lagemaat LN, Mager DL (2006). Retroviral elements and their hosts: insertional mutagenesis in the mouse germ line. PLoS Genet.

[76] Cordaux R, Batzer MA (2009). The impact of retrotransposons on human genome evolution. Nat Rev Genet.

[77] Lamprecht B, Walter K, Kreher S, Kumar R, Hummel M, Lenze D (2010). Derepression of an endogenous long terminal repeat activates the CSF1R proto-oncogene in human lymphoma. Nat Med.

[78] Kassiotis G (2014). Endogenous retroviruses and the development of cancer. J Immunol.

[79] Katoh I, Kurata S (2013). Association of endogenous retroviruses and long terminal repeats with human disorders. Frontiers Oncol.

[80] Gonzalez-Hernandez MJ, Cavalcoli JD, Sartor MA, Contreras-Galindo R, Meng F, Dai M (2014). Regulation of the human endogenous retrovirus K (HML-2) transcriptome by the HIV-1 Tat protein. J Virol.

[81] Brinzevich D, Young GR, Sebra R, Ayllon J, Maio SM, Deikus G (2014). HIV-1 interacts with human endogenous retrovirus K (HML-2) envelopes derived from human primary lymphocytes. J Virol.

[82] Michaud HA, de Mulder M, SenGupta D, Deeks SG, Martin JN, Pilcher CD (2014). Trans-activation, post-transcriptional maturation, and induction of antibodies to HERV-K (HML-2) envelope transmembrane protein in HIV-1 infection. Retrovirology.

[83] Manghera M, Douville RN (2013). Endogenous retrovirus-K promoter: a landing strip for inflammatory transcription factors?. Retrovirology.

[84] Bodega B, Orlando V (2014). Repetitive elements dynamics in cell identity programming, maintenance and disease. Curr Opin Cell Biol.

[85] Guffanti G, Gaudi S, Fallon JH, Sobell J, Potkin SG, Pato C (2014). Transposable elements and psychiatric disorders. Am J Med Genet B Neuropsychiatr Genet.

[86] Reilly MT, Faulkner GJ, Dubnau J, Ponomarev I, Gage FH (2013). The role of transposable elements in health and diseases of the central nervous system. J Neurosci.

[87] Hancks DC, Kazazian HH (2012). Active human retrotransposons: variation and disease. Curr Opin Genet Dev.

[88] St Laurent G, Hammell N, McCaffrey TA (2010). A LINE-1 component to human aging: do LINE elements exact a longevity cost for evolutionary advantage?. Mech Ageing Dev.

[89] Wissing S, Montano M, Garcia-Perez JL, Moran JV, Greene WC (2011). Endogenous APOBEC3B restricts LINE-1 retrotransposition in transformed cells and human embryonic stem cells. J Biol Chem.

[90] Aravin AA, Sachidanandam R, Girard A, Fejes-Toth K, Hannon GJ (2007). Developmentally regulated piRNA clusters implicate MILI in transposon control. Science.

[91] Volkman HE, Stetson DB (2014). The enemy within: endogenous retroelements and autoimmune disease. Nat Immunol.

[92] Leung DC, Lorincz MC (2012). Silencing of endogenous retroviruses: when and why do histone marks predominate?. Trends Biochem Sci.

[93] Wolf D, Goff SP (2008). Host restriction factors blocking retroviral replication. Annu Rev Genet.

[94] Wolf G, Nielsen AL, Mikkelsen JG, Pedersen FS (2013). Epigenetic marking and repression of porcine endogenous retroviruses. J Gen Virol.

[95] Yoder JA, Walsh CP, Bestor TH (1997). Cytosine methylation and the ecology of intragenomic parasites. Trends Genet.

[96] Jaenisch R, Schnieke A, Harbers K (1985). Treatment of mice with 5-azacytidine efficiently activates silent retroviral genomes in different tissues. Proc Natl Acad Sci U S A.

[97] Walsh CP, Chaillet JR, Bestor TH (1998). Transcription of IAP endogenous retroviruses is constrained by cytosine methylation. Nat Genet.

[98] Mikkelsen TS, Ku M, Jaffe DB, Issac B, Lieberman E, Giannoukos G (2007). Genome-wide maps of chromatin state in pluripotent and lineage-committed cells. Nature.

[99] Karimi MM, Goyal P, Maksakova IA, Bilenky M, Leung D, Tang JX (2011). DNA methylation and SETDB1/H3K9me3 regulate predominantly distinct sets of genes, retroelements, and chimeric transcripts in mESCs. Cell Stem Cell.

[100] Matsui T, Leung D, Miyashita H, Maksakova IA, Miyachi H, Kimura H (2010). Proviral silencing in embryonic stem cells requires the histone methyltransferase ESET. Nature.

[101] Rowe HM, Trono D (2011). Dynamic control of endogenous retroviruses during development. Virology.

[102] Saito K, Siomi MC (2010). Small RNA-mediated quiescence of transposable elements in animals. Dev Cell.

[103] Yamanaka S, Mehta S, Reyes-Turcu FE, Zhuang F, Fuchs RT, Rong Y (2013). RNAi triggered by specialized machinery silences developmental genes and retrotransposons. Nature.

[104] Law JA, Jacobsen SE (2010). Establishing, maintaining and modifying DNA methylation patterns in plants and animals. Nat Rev Genet.

[105] Dumesic PA, Madhani HD (2014). Recognizing the enemy within: licensing RNA-guided genome defense. Trends Biochem Sci.

[106] Marchetto MC, Narvaiza I, Denli AM, Benner C, Lazzarini TA, Nathanson JL (2013). Differential L1 regulation in pluripotent stem cells of humans and apes. Nature.

[107] Carmell MA, Girard A, van de Kant HJ, Bourc’his D, Bestor TH, de Rooij DG (2007). MIWI2 is essential for spermatogenesis and repression of transposons in the mouse male germline. Dev Cell.

[108] De Fazio S, Bartonicek N, Di Giacomo M, Abreu-Goodger C, Sankar A, Funaya C (2011). The endonuclease activity of Mili fuels piRNA amplification that silences LINE1 elements. Nature.

[109] Barklis E, Mulligan RC, Jaenisch R (1986). Chromosomal position or virus mutation permits retrovirus expression in embryonal carcinoma cells. Cell.

[110] Loh TP, Sievert LL, Scott RW (1987). Proviral sequences that restrict retroviral expression in mouse embryonal carcinoma cells. Mol Cell Biol.

[111] Wolf D, Goff SP (2007). TRIM28 mediates primer binding site-targeted silencing of murine leukemia virus in embryonic cells. Cell.

[112] Wolf D, Goff SP (2009). Embryonic stem cells use ZFP809 to silence retroviral DNAs. Nature.

[113] Rowe HM, Jakobsson J, Mesnard D, Rougemont J, Reynard S, Aktas T (2010). KAP1 controls endogenous retroviruses in embryonic stem cells. Nature.

[114] Fasching L, Kapopoulou A, Sachdeva R, Petri R, Jonsson ME, Manne C (2015). TRIM28 represses transcription of endogenous retroviruses in neural progenitor cells. Cell reports.

[115] Collins PL, Kyle KE, Egawa T, Shinkai Y, Oltz EM (2015). The histone methyltransferase SETDB1 represses endogenous and exogenous retroviruses in B lymphocytes. Proc Natl Acad Sci U S A.

[116] Tan X, Xu X, Elkenani M, Smorag L, Zechner U, Nolte J (2013). Zfp819, a novel KRAB-zinc finger protein, interacts with KAP1 and functions in genomic integrity maintenance of mouse embryonic stem cells. Stem Cell Res.

[117] Reynolds L, Ullman C, Moore M, Isalan M, West MJ, Clapham P (2003). Repression of the HIV-1 5’ LTR promoter and inhibition of HIV-1 replication by using engineered zinc-finger transcription factors. Proc Natl Acad Sci U S A.

[118] Segal DJ, Goncalves J, Eberhardy S, Swan CH, Torbett BE, Li X (2004). Attenuation of HIV-1 replication in primary human cells with a designed zinc finger transcription factor. J Biol Chem.

[119] Eberhardy SR, Goncalves J, Coelho S, Segal DJ, Berkhout B, Barbas CF (2006). Inhibition of human immunodeficiency virus type 1 replication with artificial transcription factors targeting the highly conserved primer-binding site. J Virol.

[120] Carlson KA, Leisman G, Limoges J, Pohlman GD, Horiba M, Buescher J (2004). Molecular characterization of a putative antiretroviral transcriptional factor, OTK18. J Immunol.

[121] Horiba M, Martinez LB, Buescher JL, Sato S, Limoges J, Jiang Y (2007). OTK18, a zinc-finger protein, regulates human immunodeficiency virus type 1 long terminal repeat through two distinct regulatory regions. J Gen Virol.

[122] Nishitsuji H, Abe M, Sawada R, Takaku H (2012). ZBRK1 represses HIV-1 LTR-mediated transcription. FEBS Lett.

[123] Nishitsuji H, Sawada L, Sugiyama R, Takaku H (2015). ZNF10 inhibits HIV-1 LTR activity through interaction with NF-kappaB and Sp1 binding motifs. FEBS Lett.

[124] Gifford RJ, Katzourakis A, Tristem M, Pybus OG, Winters M, Shafer RW (2008). A transitional endogenous lentivirus from the genome of a basal primate and implications for lentivirus evolution. Proc Natl Acad Sci U S A.

[125] Worobey M, Telfer P, Souquiere S, Hunter M, Coleman CA, Metzger MJ (2010). Island biogeography reveals the deep history of SIV. Science.

[126] Paiva A, Casseb J (2015). Origin and prevalence of human T-lymphotropic virus type 1 (HTLV-1) and type 2 (HTLV-2) among indigenous populations in the Americas. Rev Inst Med Trop Sao Paulo.

[127] Mahieux R, Gessain A (2011). HTLV-3/STLV-3 and HTLV-4 viruses: discovery, epidemiology, serology and molecular aspects. Viruses.

[128] Buescher JL, Duan F, Sun J, Price RW, Ikezu T (2008). OTK18 levels in plasma and cerebrospinal fluid correlate with viral load and CD8 T-cells in normal and AIDS patients. J Neuroimmune Pharmacol.

[129] Carlson KA, Limoges J, Pohlman GD, Poluektova LY, Langford D, Masliah E (2004). OTK18 expression in brain mononuclear phagocytes parallels the severity of HIV-1 encephalitis. J Neuroimmunol.

[130] Yun J, Lee WH (2003). Degradation of transcription repressor ZBRK1 through the ubiquitin-proteasome pathway relieves repression of Gadd45a upon DNA damage. Mol Cell Biol.

[131] Lee YK, Thomas SN, Yang AJ, Ann DK (2007). Doxorubicin down-regulates Kruppel-associated box domain-associated protein 1 sumoylation that relieves its transcription repression on p21WAF1/CIP1 in breast cancer MCF-7 cells. J Biol Chem.

[132] Furuta S, Wang JM, Wei S, Jeng YM, Jiang X, Gu B (2006). Removal of BRCA1/CtIP/ZBRK1 repressor complex on ANG1 promoter leads to accelerated mammary tumor growth contributed by prominent vasculature. Cancer Cell.

[133] Lin LF, Chuang CH, Li CF, Liao CC, Cheng CP, Cheng TL (2010). ZBRK1 acts as a metastatic suppressor by directly regulating MMP9 in cervical cancer. Cancer Res.

[134] Yu EJ, Kim SH, Kim MJ, Seo WY, Song KA, Kang MS (2013). SUMOylation of ZFP282 potentiates its positive effect on estrogen signaling in breast tumorigenesis. Oncogene.

[135] Yeo SY, Ha SY, Yu EJ, Lee KW, Kim JH, Kim SH (2014). ZNF282 (Zinc finger protein 282), a novel E2F1 co-activator, promotes esophageal squamous cell carcinoma. Oncotarget.

[136] Gilling CE, Carlson KA (2009). The effect of OTK18 upregulation in U937 cells on neuronal survival. In vitro cellular Developmental biology Animal.

[137] Engler P, Doglio LT, Bozek G, Storb U (1998). A cis-acting element that directs the activity of the murine methylation modifier locus Ssm1. Proc Natl Acad Sci U S A.

[138] Padjen K, Ratnam S, Storb U (2005). DNA methylation precedes chromatin modifications under the influence of the strain-specific modifier Ssm1. Mol Cell Biol.

[139] Ratnam S, Engler P, Bozek G, Mao L, Podlutsky A, Austad S (2014). Identification of Ssm1b, a novel modifier of DNA methylation, and its expression during mouse embryogenesis. Development.

[140] Weng A, Magnuson T, Storb U (1995). Strain-specific transgene methylation occurs early in mouse development and can be recapitulated in embryonic stem cells. Development.

[141] Aiewsakun P, Katzourakis A (2015). Endogenous viruses: Connecting recent and ancient viral evolution. Virology.

[142] Pelisson A, Mejlumian L, Robert V, Terzian C, Bucheton A (2002). Drosophila germline invasion by the endogenous retrovirus gypsy: involvement of the viral env gene. Insect Biochem Mol Biol.

[143] Jolly C (2011). Cell-to-cell transmission of retroviruses: Innate immunity and interferon-induced restriction factors. Virology.

[144] Flajnik MF, Kasahara M (2010). Origin and evolution of the adaptive immune system: genetic events and selective pressures. Nat Rev Genet.

[145] Collins T, Stone JR, Williams AJ (2001). All in the family: the BTB/POZ, KRAB, and SCAN domains. Mol Cell Biol.

[146] Ivanov D, Stone JR, Maki JL, Collins T, Wagner G (2005). Mammalian SCAN domain dimer is a domain-swapped homolog of the HIV capsid C-terminal domain. Mol Cell.

[147] Kingston RL, Vogt VM (2005). Domain swapping and retroviral assembly. Mol Cell.

[148] Barde I, Laurenti E, Verp S, Groner AC, Towne C, Padrun V (2009). Regulation of episomal gene expression by KRAB/KAP1-mediated histone modifications. J Virol.

[149] Allouch A, Di Primio C, Alpi E, Lusic M, Arosio D, Giacca M (2011). The TRIM family protein KAP1 inhibits HIV-1 integration. Cell Host Microbe.

[150] Lukic S, Nicolas JC, Levine AJ (2014). The diversity of zinc-finger genes on human chromosome 19 provides an evolutionary mechanism for defense against inherited endogenous retroviruses. Cell Death Differ.

[151] Cohen CJ, Lock WM, Mager DL (2009). Endogenous retroviral LTRs as promoters for human genes: a critical assessment. Gene.

[152] Beyer U, Moll-Rocek J, Moll UM, Dobbelstein M (2011). Endogenous retrovirus drives hitherto unknown proapoptotic p63 isoforms in the male germ line of humans and great apes. Proc Natl Acad Sci U S A.

[153] Sundaram V, Cheng Y, Ma Z, Li D, Xing X, Edge P (2014). Widespread contribution of transposable elements to the innovation of gene regulatory networks. Genome Res.

[154] Lynch VJ, Nnamani MC, Kapusta A, Brayer K, Plaza SL, Mazur EC (2015). Ancient Transposable Elements Transformed the Uterine Regulatory Landscape and Transcriptome during the Evolution of Mammalian Pregnancy. Cell reports.

[155] Wang J, Xie G, Singh M, Ghanbarian AT, Rasko T, Szvetnik A (2014). Primate-specific endogenous retrovirus-driven transcription defines naive-like stem cells. Nature.

[156] Robbez-Masson L, Rowe HM (2015). Retrotransposons shape species-specific embryonic stem cell gene expression. Retrovirology.

[157] Rowe HM, Kapopoulou A, Corsinotti A, Fasching L, Macfarlan TS, Tarabay Y (2013). TRIM28 repression of retrotransposon-based enhancers is necessary to preserve transcriptional dynamics in embryonic stem cells. Genome Res.

[158] Plamondon JA, Harris MJ, Mager DL, Gagnier L, Juriloff DM (2011). The clf2 gene has an epigenetic role in the multifactorial etiology of cleft lip and palate in the A/WySn mouse strain. Birth Defects Res A Clin Mol Teratol.

[159] Gage FH (2000). Mammalian neural stem cells. Science.

[160] Li X, Ito M, Zhou F, Youngson N, Zuo X, Leder P (2008). A maternal-zygotic effect gene, Zfp57, maintains both maternal and paternal imprints. Dev Cell.

[161] Mackay DJ, Callaway JL, Marks SM, White HE, Acerini CL, Boonen SE (2008). Hypomethylation of multiple imprinted loci in individuals with transient neonatal diabetes is associated with mutations in ZFP57. Nat Genet.

[162] Strogantsev R, Krueger F, Yamazawa K, Shi H, Gould P, Goldman-Roberts M (2015). Allele-specific binding of ZFP57 in the epigenetic regulation of imprinted and non-imprinted monoallelic expression. Genome Biol.

[163] Cowley M, Oakey RJ (2010). Retrotransposition and genomic imprinting. Briefings in functional genomics.

[164] Krebs CJ, Larkins LK, Price R, Tullis KM, Miller RD, Robins DM (2003). Regulator of sex-limitation (Rsl) encodes a pair of KRAB zinc-finger genes that control sexually dimorphic liver gene expression. Genes Dev.

[165] Nelson DR, Zeldin DC, Hoffman SM, Maltais LJ, Wain HM, Nebert DW (2004). Comparison of cytochrome P450 (CYP) genes from the mouse and human genomes, including nomenclature recommendations for genes, pseudogenes and alternative-splice variants. Pharmacogenetics.

[166] Bojkowska K, Aloisio F, Cassano M, Kapopoulou A, Santoni De Sio F, Zangger N (2012). Liver-specific ablation of Kruppel-associated box-associated protein 1 in mice leads to male-predominant hepatosteatosis and development of liver adenoma. Hepatology.

[167] Thomas JH (2007). Rapid birth-death evolution specific to xenobiotic cytochrome P450 genes in vertebrates. PLoS Genet.

[168] Stavenhagen JB, Robins DM (1988). An ancient provirus has imposed androgen regulation on the adjacent mouse sex-limited protein gene. Cell.

[169] Loreni F, Stavenhagen J, Kalff M, Robins DM (1988). A complex androgen-responsive enhancer resides 2 kilobases upstream of the mouse Slp gene. Mol Cell Biol.

[170] Krebs CJ, Schultz DC, Robins DM (2012). The KRAB zinc finger protein RSL1 regulates sex- and tissue-specific promoter methylation and dynamic hormone-responsive chromatin configuration. Mol Cell Biol.

[171] Krebs CJ, Larkins LK, Khan SM, Robins DM (2005). Expansion and diversification of KRAB zinc-finger genes within a cluster including Regulator of sex-limitation 1 and 2. Genomics.

[172] Baker CL, Kajita S, Walker M, Saxl RL, Raghupathy N, Choi K (2015). PRDM9 drives evolutionary erosion of hotspots in Mus musculus through haplotype-specific initiation of meiotic recombination. PLoS Genet.

[173] Ptak SE, Hinds DA, Koehler K, Nickel B, Patil N, Ballinger DG (2005). Fine-scale recombination patterns differ between chimpanzees and humans. Nat Genet.

[174] Winckler W, Myers SR, Richter DJ, Onofrio RC, McDonald GJ, Bontrop RE (2005). Comparison of fine-scale recombination rates in humans and chimpanzees. Science.

[175] Coop G, Wen X, Ober C, Pritchard JK, Przeworski M (2008). High-resolution mapping of crossovers reveals extensive variation in fine-scale recombination patterns among humans. Science.

[176] Segurel L, Leffler EM, Przeworski M (2011). The case of the fickle fingers: how the PRDM9 zinc finger protein specifies meiotic recombination hotspots in humans. PLoS Biol.

[177] Parvanov ED, Petkov PM, Paigen K (2010). Prdm9 controls activation of mammalian recombination hotspots. Science.

[178] Baudat F, Buard J, Grey C, Fledel-Alon A, Ober C, Przeworski M (2010). PRDM9 is a major determinant of meiotic recombination hotspots in humans and mice. Science.

[179] Berg IL, Neumann R, Lam KW, Sarbajna S, Odenthal-Hesse L, May CA (2010). PRDM9 variation strongly influences recombination hot-spot activity and meiotic instability in humans. Nat Genet.

[180] Schwartz JJ, Roach DJ, Thomas JH, Shendure J (2014). Primate evolution of the recombination regulator PRDM9. Nat Commun.

[181] Oliver PL, Goodstadt L, Bayes JJ, Birtle Z, Roach KC, Phadnis N (2009). Accelerated evolution of the Prdm9 speciation gene across diverse metazoan taxa. PLoS Genet.

[182] Thomas JH, Emerson RO, Shendure J (2009). Extraordinary molecular evolution in the PRDM9 fertility gene. PLoS One.

[183] Myers S, Bowden R, Tumian A, Bontrop RE, Freeman C, MacFie TS (2010). Drive against hotspot motifs in primates implicates the PRDM9 gene in meiotic recombination. Science.

[184] Myers S, Bottolo L, Freeman C, McVean G, Donnelly P (2005). A fine-scale map of recombination rates and hotspots across the human genome. Science.

[185] Smit AF (1993). Identification of a new, abundant superfamily of mammalian LTR-transposons. Nucleic Acids Res.

[186] Billings T, Parvanov ED, Baker CL, Walker M, Paigen K, Petkov PM (2013). DNA binding specificities of the long zinc-finger recombination protein PRDM9. Genome Biol.

[187] Brayer KJ, Kulshreshtha S, Segal DJ (2008). The protein-binding potential of C2H2 zinc finger domains. Cell Biochem Biophys.

[188] Iuchi S (2001). Three classes of C2H2 zinc finger proteins. Cell Mol Life Sci.

[189] He Q, Johnston J, Zeitlinger J (2015). ChIP-nexus enables improved detection of in vivo transcription factor binding footprints. Nat Biotechnol.

[190] Rhee HS, Pugh BF (2011). Comprehensive genome-wide protein-DNA interactions detected at single-nucleotide resolution. Cell.

[191] Rizkallah R, Hurt MM (2009). Regulation of the transcription factor YY1 in mitosis through phosphorylation of its DNA-binding domain. Mol Biol Cell.

[192] Chien HC, Wang HY, Su YN, Lai KY, Lu LC, Chen PC (2012). Targeted disruption in mice of a neural stem cell-maintaining, KRAB-Zn finger-encoding gene that has rapidly evolved in the human lineage. PLoS One.

[193] Lockwood SH, Guan A, Yu AS, Zhang C, Zykovich A, Korf I (2014). The functional significance of common polymorphisms in zinc finger transcription factors. G3.

[194] Carbone L, Harris RA, Gnerre S, Veeramah KR, Lorente-Galdos B, Huddleston J (2014). Gibbon genome and the fast karyotype evolution of small apes. Nature.

[195] Lupan I, Bulzu P, Popescu O, Damert A (2015). Lineage specific evolution of the VNTR composite retrotransposon central domain and its role in retrotransposition of gibbon LAVA elements. BMC Genomics.

[196] Carbone L, Harris RA, Mootnick AR, Milosavljevic A, Martin DI, Rocchi M (2012). Centromere remodeling in Hoolock leuconedys (Hylobatidae) by a new transposable element unique to the gibbons. Genome Biol Evol.

[197] Edgar RC (2004). MUSCLE: multiple sequence alignment with high accuracy and high throughput. Nucleic Acids Res.

[198] Tamura K, Stecher G, Peterson D, Filipski A, Kumar S (2013). MEGA6: Molecular Evolutionary Genetics Analysis version 6.0. Mol Biol Evol.

[199] Grant CE, Bailey TL, Noble WS (2011). FIMO: scanning for occurrences of a given motif. Bioinformatics.

[200] Shen L, Shao N, Liu X, Nestler E (2014). ngs.plot: Quick mining and visualization of next-generation sequencing data by integrating genomic databases. BMC Genomics.

